# Decoding Mammalian Ribosome-mRNA States by Translational GTPase Complexes

**DOI:** 10.1016/j.cell.2016.10.046

**Published:** 2016-11-17

**Authors:** Sichen Shao, Jason Murray, Alan Brown, Jack Taunton, V. Ramakrishnan, Ramanujan S. Hegde

**Affiliations:** 1 MRC-LMB, Francis Crick Avenue, Cambridge CB2 0QH, UK; 2 Department of Cellular and Molecular Pharmacology, University of California, San Francisco, San Francisco, CA 94158, USA

**Keywords:** mammalian ribosome, protein translation, mRNA decoding, translational GTPase, cryo-EM

## Abstract

In eukaryotes, accurate protein synthesis relies on a family of translational GTPases that pair with specific decoding factors to decipher the mRNA code on ribosomes. We present structures of the mammalian ribosome engaged with decoding factor⋅GTPase complexes representing intermediates of translation elongation (aminoacyl-tRNA⋅eEF1A), termination (eRF1⋅eRF3), and ribosome rescue (Pelota⋅Hbs1l). Comparative analyses reveal that each decoding factor exploits the plasticity of the ribosomal decoding center to differentially remodel ribosomal proteins and rRNA. This leads to varying degrees of large-scale ribosome movements and implies distinct mechanisms for communicating information from the decoding center to each GTPase. Additional structural snapshots of the translation termination pathway reveal the conformational changes that choreograph the accommodation of decoding factors into the peptidyl transferase center. Our results provide a structural framework for how different states of the mammalian ribosome are selectively recognized by the appropriate decoding factor⋅GTPase complex to ensure translational fidelity.

## Highlights

• Cryo-EM structures of elongating, terminating, and stalled mammalian ribosomes
• Eukaryotic-specific elements contribute to stringent sense and stop codon decoding
• Pelota engages stalled ribosomes by destabilizing mRNA in the mRNA channel
• Decoding complexes communicate recognition to GTPase activation in different ways

## Graphical Abstract

**Figure:**
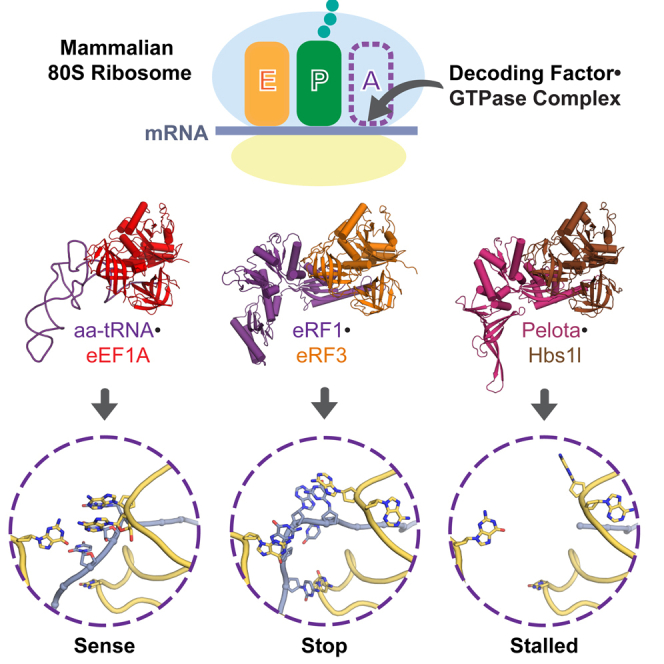

## Teaser

The individual decoding factor⋅GTPase complexes involved in protein synthesis differentially remodel local protein and RNA elements on ribosomes to ensure translation fidelity.

## Introduction

Successful protein synthesis by ribosomes requires amino acids to be incorporated correctly during polypeptide elongation, translation to terminate at precise points, and quality control pathways to be engaged when translation is interrupted (Dever and Green, 2012). In eukaryotes, each of these events is mediated by specific factors (collectively termed as decoding factors in this study) that are delivered to the A site of the ribosome by a specialized member of a subfamily of translational GTPases. Members of this GTPase subfamily are structurally homologous but have non-redundant functions (Dever and Green, 2012): eEF1A delivers aminoacyl (aa)-tRNAs to sense codons; eRF3 delivers eRF1 to stop codons; and Hbs1l delivers Pelota (Dom34 in yeast) to stalled ribosomes. After delivery, the specificity of each decoding factor is inspected at the ribosomal decoding center before being accepted into the catalytic peptidyl transferase center (PTC) of the ribosome. Acceptance of each decoding factor by the ribosome has distinct and irreversible consequences: amino acid addition by aa-tRNA, translation termination by eRF1, and the initiation of mRNA and protein quality-control pathways by Pelota. Therefore, accurate decoding of the transcriptome and maintenance of protein homeostasis relies on decoding factor⋅GTPase complexes recognizing the appropriate ribosome-mRNA complex.

Our mechanistic understanding of decoding derives primarily from functional and structural studies of sense codon recognition by aa-tRNAs and the bacterial eEF1A homolog, EF-Tu (Voorhees and Ramakrishnan, 2013). The accuracy of accepting the correct aa-tRNA is enhanced by a two-step mechanism that exploits the interactions at the decoding center twice. GTP hydrolysis by EF-Tu irreversibly separates an initial selection step from a secondary kinetic proofreading step (Blanchard et al., 2004). During initial selection, aa-tRNA in complex with EF-Tu⋅GTP samples ribosomes in a configuration in which the aminoacyl group of the aa-tRNA is held by EF-Tu to prevent premature engagement with the PTC (Schmeing et al., 2009). Cognate interactions between aa-tRNA and mRNA at the ribosomal decoding center are communicated to EF-Tu to activate GTP hydrolysis (Pape et al., 1998, Ogle et al., 2001, Ogle et al., 2002), which ultimately leads to the dissociation of EF-Tu⋅GDP from the ribosomal complex (Schmeing et al., 2009). This frees the aa-tRNA to “accommodate” into the ribosomal PTC, a rate-limiting step that relies on the stability of the codon-anticodon interactions at the ribosomal decoding center (Pape et al., 1998).

Important differences from the paradigm established by aa-tRNA⋅EF-Tu probably exist for eukaryotic decoding factor⋅translational GTPase complexes to account for higher translation accuracy (Kramer et al., 2010), the evolutionary divergence of the mammalian ribosome, and the eukaryotic expansion of the translational GTPase family to deliver non-tRNA factors to the ribosomal A site (Atkinson et al., 2008). Biochemical studies and moderate-resolution structures of several eukaryotic decoding complexes have revealed insights into conserved and distinct features of eukaryotic decoding complexes (Becker et al., 2011, Dever and Green, 2012, Shoemaker and Green, 2012, Taylor et al., 2012, des Georges et al., 2014, Preis et al., 2014). However, the molecular interactions that accompany initial selection, communicate information from the decoding center to each GTPase, and mediate decoding factor accommodation in each case remain incompletely understood. Using high-resolution electron cryomicroscopy (cryo-EM), we analyze the molecular basis of specificity at the decoding center for each mammalian decoding factor⋅translational GTPase complex, compare potential GTPase activation mechanisms, and describe the conformational changes governing the accommodation of decoding factors. These results provide new insights into how these related complexes are able to make discriminatory interactions to recognize the appropriate ribosome-mRNA substrates to maintain overall translational fidelity.

## Results and Discussion

### Cryo-EM Structures of Eukaryotic Translational Decoding Complexes

**Figure S1:**
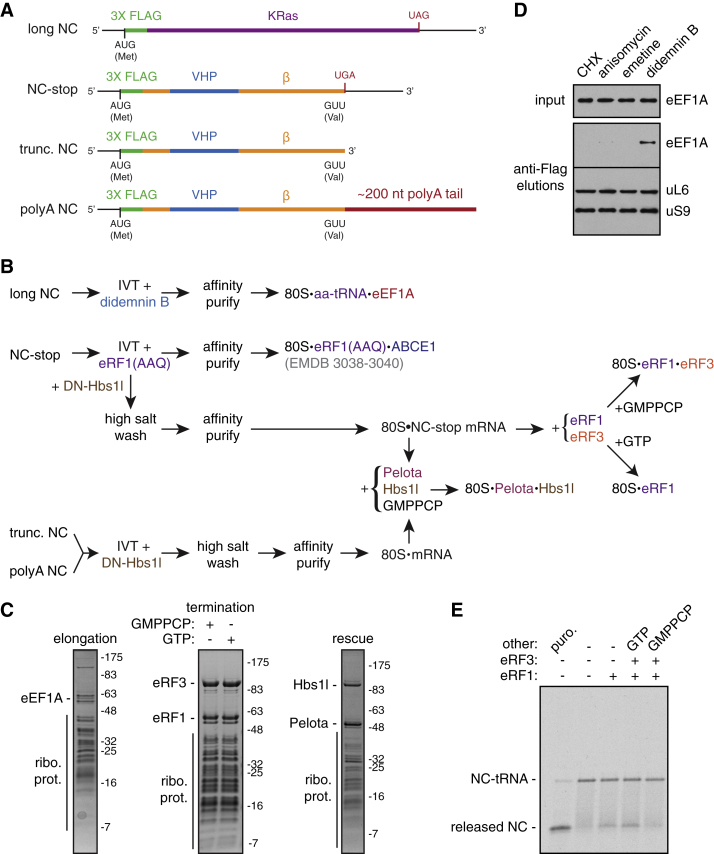
Isolation of Translational Decoding Complexes for Cryo-EM, Related to Figure 1 (A) Schematic of the mRNA constructs used for in vitro translation and isolation of ribosome-nascent chain complexes (RNCs). The start codon (AUG), stop codon (UAG or UGA), and coding regions for the 3X Flag tag (green), the autonomously-folding villin headpiece (VHP) domain (blue), the cytosolic portion of Sec61β (orange), and KRas (purple) are indicated. (B) Experimental strategies for isolating the indicated RNCs from in vitro translation (IVT) reactions. (C) SDS-PAGE and Coomassie staining of isolated RNCs representing the elongation complex (80S⋅aa-tRNA⋅eEF1A); pre-accommodated (80S⋅eRF1⋅eRF3) or accommodated (80S⋅eRF1) termination complexes; and rescue complex (80S⋅Pelota⋅Hbs1l) reconstituted with a truncated mRNA (see panel A). Copurified, exogenously-added, and ribosomal (ribo. prot.) proteins are indicated. (D) The long NC construct (see panel A) was translated in vitro in rabbit reticulocyte lysate (RRL) with the indicated translational inhibitors added at the following concentrations: 50 μg/mL cycloheximide (CHX), 10 μM anisomycin, 200 μM emetine, and 50 μM didemnin B. The translation reactions were affinity purified via the 3X Flag tag on the nascent chain. The elutions and inputs were analyzed by SDS-PAGE and immunoblotting for the indicated proteins, revealing that didemnin B specifically traps eEF1A on the isolated RNCs. (E) The NC-stop construct was translated in vitro in RRL in the presence of ^35^S-methionine and mutant eRF1(AAQ) to trap RNCs with the UGA stop codon in the A site. The RNCs were isolated under high salt conditions and subjected to affinity purification via the 3X Flag tag on the nascent chain. The isolated RNCs were incubated with 1 mM puromycin or recombinant wild-type eRF1, wild-type eRF3, and 0.5 mM GMPPCP or GTP as indicated, and then directly analyzed by SDS-PAGE and autoradiography. The bands corresponding to ribosome-associated nascent chain-tRNA (NC-tRNA) and released nascent chains (NC) are indicated. This demonstrates the functionality of the components of the reconstituted termination complex in mediating the release of the nascent chain, which is inhibited by the nonhydrolyzable GTP analog, GMPPCP.

**Figure S2:**
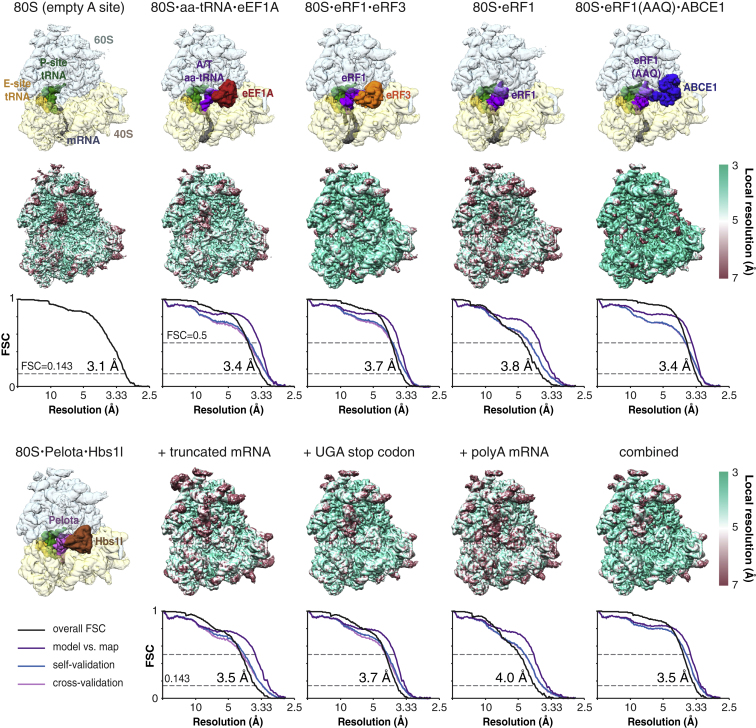
Quality of Cryo-EM Maps and Models, Related to Figure 1 The EM map for each isolated RNC complex is shown colored according to individual factors (top row) or by local resolution (second row). Below each local resolution map are Fourier shell correlation (FSC) curves calculated between independent half maps (black), and calculated between the refined model and final map (purple), and with the self (blue) and cross-validated (magenta) correlations for each complex. The nominal resolution estimated from the map-to-map correlation at FSC = 0.143 is reported and agrees well with the model-to-map correlation at FSC = 0.5. The 80S⋅eRF1(AAQ)⋅ABCE1 map was generated by combining all of the datasets from (Brown et al., 2015b) to analyze eRF1 conformational changes during the termination pathway (see Figures 7 and S7).

Translational decoding complexes (here defined as the elongation complex, 80S⋅aa-tRNA⋅eEF1A; the termination complex, 80S⋅eRF1⋅eRF3; and the rescue complex, 80S⋅Pelota⋅Hbs1l) are transient states that either rapidly dissociate or progress to an accommodated state upon codon recognition. We therefore developed methods to trap or assemble these complexes (Figure S1 and STAR Methods). To prepare the elongation complex, ongoing in vitro translation reactions in rabbit reticulocyte lysate of an N-terminally tagged protein were inhibited by the elongation inhibitor didemnin B (Rinehart et al., 1981), and the ribosome-nascent chains (RNCs) were affinity purified via the partially synthesized nascent polypeptide. To generate the termination complex, we programmed and affinity purified RNCs with a UGA stop codon in the A site that were reconstituted with eRF1, eRF3, and the nonhydrolyzable GTP analog GMPPCP. Rescue complexes were prepared similarly to produce RNCs containing an empty A site (generated with a truncated mRNA), or an A site occupied by either a stop codon or an AAA codon within a polyadenylated (poly(A)) tail, that were reconstituted with Pelota, Hbs1l, and GMPPCP. The structure of each complex was solved by cryo-EM to between 3.3 and 3.8 Å resolution (Figure S2; Tables S1 and S2).

**Figure 1:**
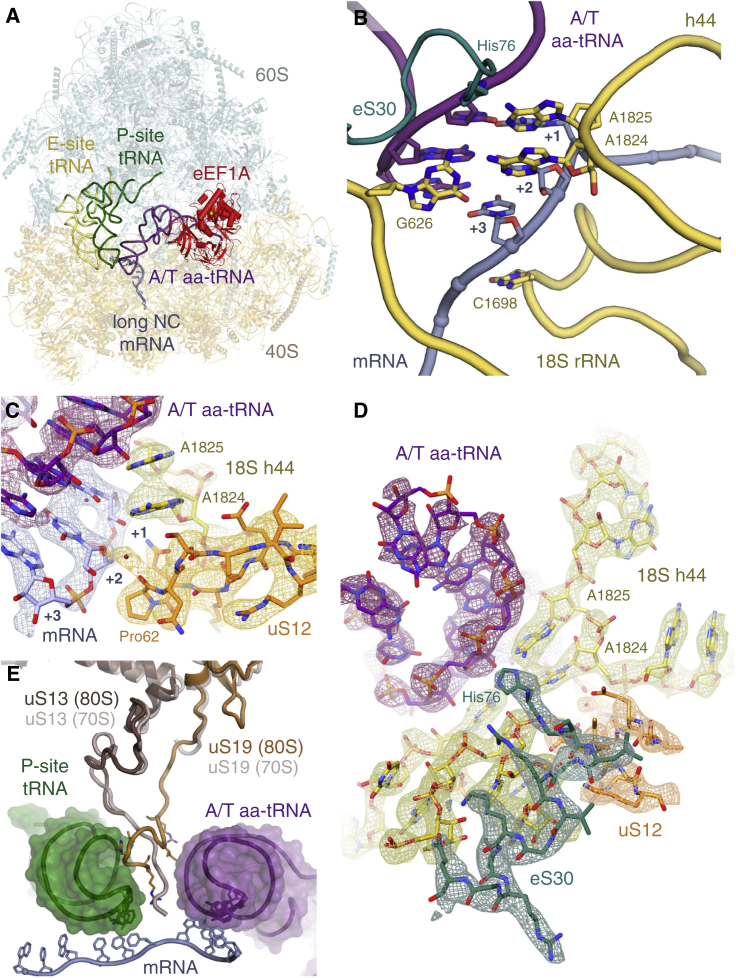
Structure of the Mammalian Elongation Complex (A) Overview of the elongation complex comprising the large (60S) and small (40S) ribosomal subunits, P- (green) and E-site (gold) tRNAs, mRNA (slate), aminoacyl-tRNA in the A/T state (aa-tRNA; purple), and eEF1A (red). (B) Decoding center of the elongation complex. eS30 (teal) and the decoding nucleotides of 18S rRNA (yellow) are indicated. (C) EM map density and models of the interactions within the decoding center of the elongation complex. Decoding nucleotides of 18S rRNA (yellow), aa-tRNA (purple), the A-site codon (+1 to +3) of mRNA (slate), and uS12 (orange) are indicated. (D) Density and models of the interaction between His76 of the N terminus of eS30 (teal) within the decoding center of the elongation complex. In panels (C) and (D), density for mRNA, tRNA, and rRNA is contoured at 9σ; density for uS12 and eS30 is contoured at 5σ. (E) The C termini of uS19 (bronze) and uS13 (brown) of the mammalian (80S) elongation complex compared to the homologous proteins in a 70S bacterial elongation complex (gray, PDB: 4V51), showing the potential interactions of the C terminus of uS19 in mammals or uS13 in bacteria with the anticodon stem loops of A/T aa-tRNA (purple) and P-site tRNA (green). See also Figures S1, S2, S3, and S4.

**Figure 2:**
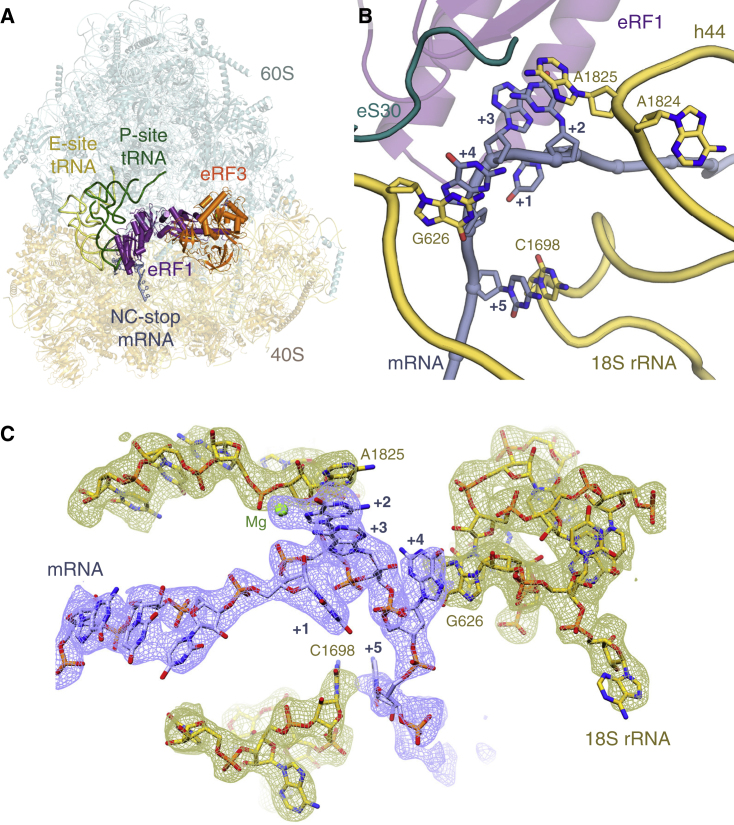
Structure of the Mammalian Termination Complex (A) Overview of the termination complex assembled with eRF1 (purple) and eRF3 (orange). (B) Decoding center of the termination complex. (C) EM map density (contoured at 6σ) and model showing interactions of the mRNA containing the UGA stop codon (slate) with rRNA elements of the decoding center (yellow).

**Figure 3:**
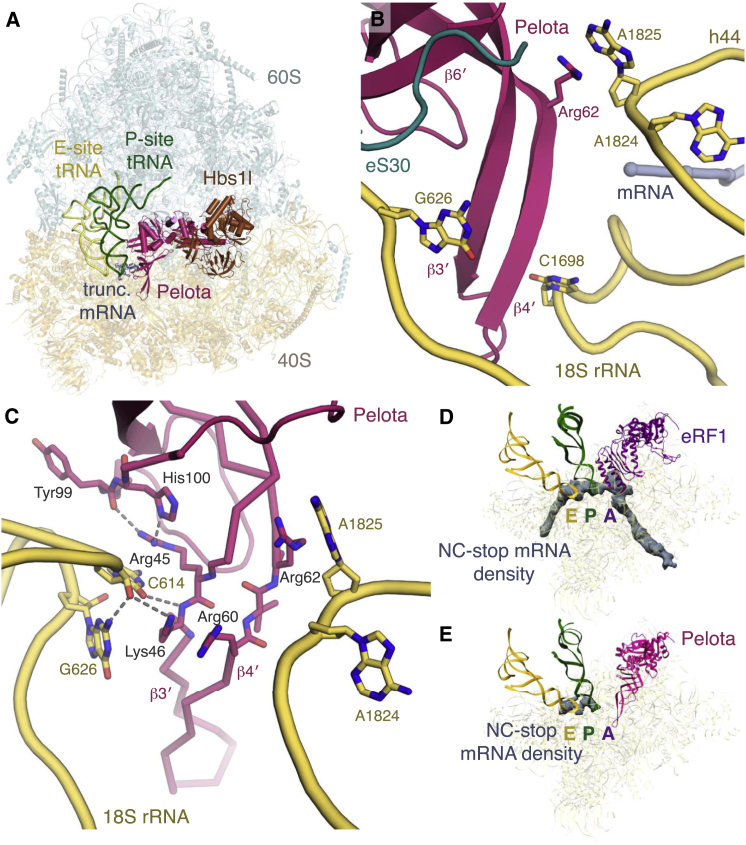
Structure of the Mammalian Rescue Complex (A) Overview of the rescue complex assembled with Pelota (pink) and Hbs1l (brown). (B) Decoding center of the rescue complex. (C) Hydrogen-bonding interactions between the β3′-β4′ loop of Pelota (pink) and 18S rRNA nucleotides (yellow). (D and E) Density corresponding to mRNA in the (D) termination or (E) rescue complexes both assembled on the same (NC-stop) mRNA stalled with the UGA stop codon in the A site. The ribosomal small subunit, P- and E-site tRNAs, and eRF1 or Pelota are indicated.

**Figure S3:**
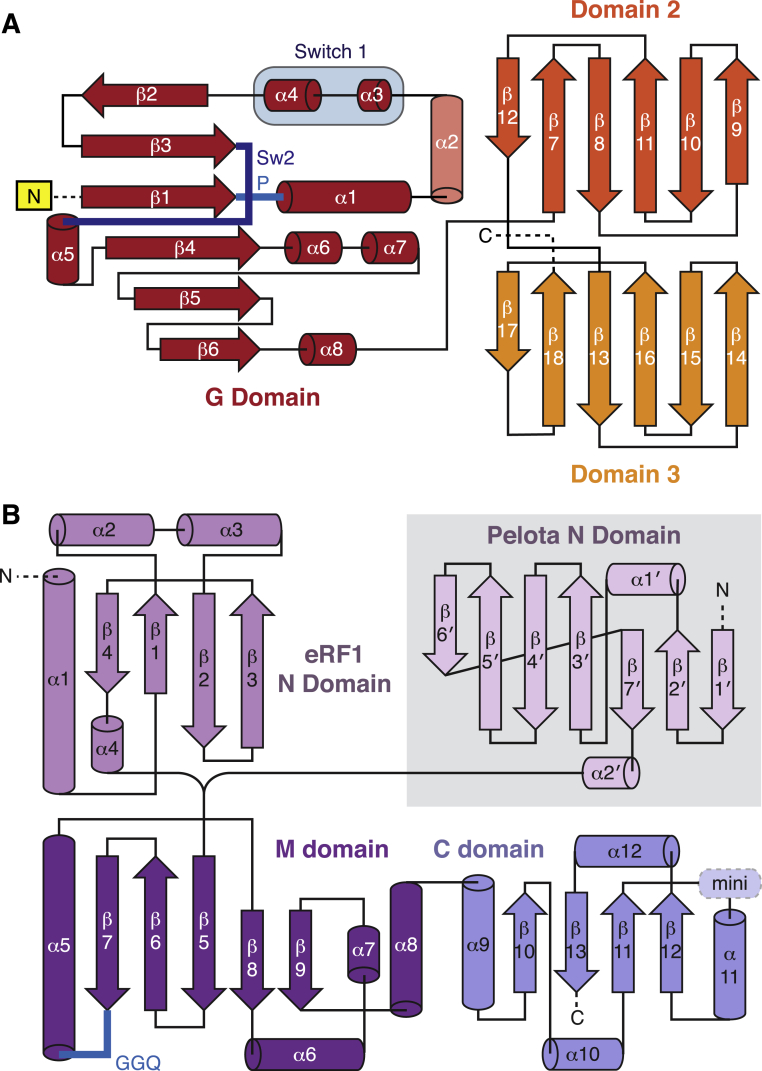
Secondary Structure Topology Diagrams of Translational GTPases and Decoding Proteins, Related to Figure 1 (A) Topology diagram of the homologous regions of translational GTPases (e.g., eEF1A, eRF3, and Hbs1l), showing the G domain (red) and the two β-barrel domains (orange and yellow). The motifs important for GTP hydrolysis (Switch 1, Switch 2 (Sw2), and P loop) are highlighted. (B) Topology diagrams of eRF1 and Pelota, showing the divergent N domains and homologous M and C domains. The locations of the loop harboring the catalytic GGQ motif (blue) and the minidomain (mini) in eRF1 are indicated.

Each complex represents an unrotated ribosome containing canonical P- and E-site tRNAs (Figures 1, 2, 3, and S2). The GTPase (G) domain and domains 2 and 3 of each GTPase (Figure S3A) were well resolved, while the highly divergent N-terminal extensions of Hbs1l and eRF3 were not visualized, presumably due to their flexibility. Each decoding factor (Figure S3B) assumes a pre-accommodated conformation: the tRNA acceptor arm or the homologous M-C domains of eRF1 or Pelota interacts with the GTPase, and the tRNA anticodon stem loop or structurally distinct N domain of eRF1 or Pelota occupies the decoding center (Figures 1, 2, and 3).

### Decoding Factor Interactions at the Ribosomal Decoding Center

#### Sense Codon Decoding in Eukaryotes

As the ribosomes in the elongation complex (Figure 1A) are stalled at different codons by didemnin B, the density for the mRNA, aa-tRNAs, and the nascent chain are averages of the species captured. Despite this, the density at the decoding center is well defined, revealing that decoding in eukaryotes shares many features with that in bacteria (Ogle et al., 2001). In particular, the decoding nucleotides A1824 and A1825 (A1492 and A1493 in bacteria) are flipped out of helix 44 (h44) of 18S rRNA. Together with G626 (G530 in bacteria) in the anti-conformation, these bases inspect the geometry of the minor groove of the codon-anticodon helix (Figure 1B) and help stabilize the A-site tRNA via hydrogen bonding. These interactions monitor Watson-Crick base-pairing at the first two codon positions (+1 and +2) while providing tolerance at the +3 wobble position.

**Figure S4:**
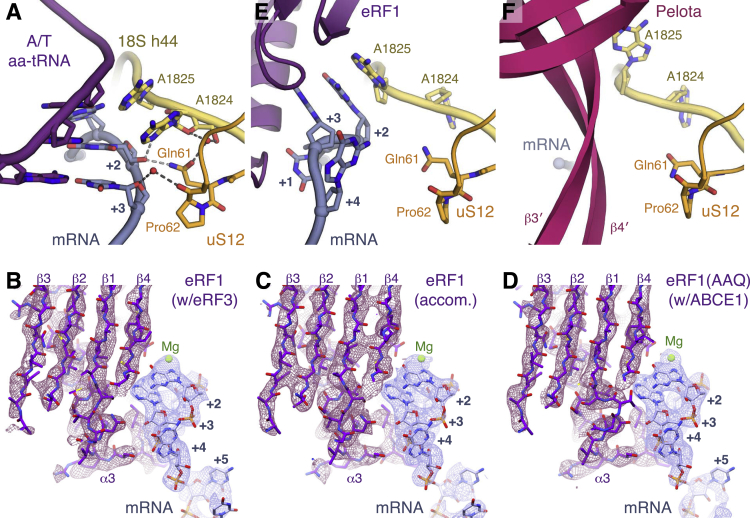
Decoding Center Interactions, Related to Figure 1 (A) Decoding center interactions of A/T aa-tRNA (purple) in the elongation complex, demonstrating how Gln61 and *cis-*Pro62 on a loop of uS12 (orange) can interact, via a water molecule or metal ion, with the mRNA (slate) backbone. Decoding nucleotides of 18S rRNA (yellow) are indicated. (B–D) EM map density and model showing that the interactions between eRF1 (purple) and stop codon mRNA (slate) remain unchanged in the (B) pre-accommodated (contoured at 8σ), (C) accommodated (contoured at 7σ), and (D) ABCE1-bound complexes (contoured at 8σ). (E and F) Decoding center interactions of (E) eRF1 (purple) in the termination complex and of (F) Pelota (pink), viewed as in panel (A).

As in bacteria (Ogle et al., 2001), the ribosomal protein uS12 projects a loop into the decoding center (Figures 1C and S4A). Gln61 (Lys44 in *E. coli*) at the apex of the loop indirectly hydrogen bonds with A1824 in its flipped-out position and with the +2 nucleotide. Pro62 adopts a conserved *cis*-peptide conformation (Noeske et al., 2015) that allows its backbone carbonyl to form a water- or metal-mediated hydrogen bond with the +3 nucleotide (Figures 1C and S4A). Additional hydrogen bonds may be introduced by environmental condition-dependent hydroxylation of Pro62 (Loenarz et al., 2014, Noeske et al., 2015). Notably, these hydrogen bonds are only with the mRNA backbone, allowing for wobble base-pairing at the +3 position.

Relative to bacterial decoding, the eukaryotic-specific ribosomal protein eS30 may enhance the stability of a correct codon-anticodon interaction. In the presence of a cognate aa-tRNA, the N terminus of eS30 becomes ordered, allowing a conserved histidine (His76) to reach into a groove between the phosphate backbone of the anticodon +1 position and the two flipped-out decoding bases to form potentially stabilizing contacts (Figures 1B and 1D). Because this groove depends on the flipped nucleotides that accompany canonical codon-anticodon base-pairing, this interaction may preferentially stabilize cognate tRNAs to enhance discrimination.

The A- and P-site tRNAs also appear to stabilize 15 residues at the C terminus of uS19 that interacts with the phosphate backbone of the P-site tRNA and may make electrostatic interactions with the A-site tRNA (Figure 1E). Similar tRNA-dependent transitions in ribosomal proteins are observed in bacteria, with the C terminus of uS13 instead of uS19 threading between the anticodon stem loops of the A- and P-site tRNAs in bacteria (Jenner et al., 2010). Deletion of the uS13 C terminus in bacteria is associated with a reduced rate of translation and less efficient tRNA selection (Faxén et al., 1994). Thus, the contacts formed by uS19, and especially by eS30, which is dependent on a cognate aa-tRNA, could increase the stability of aa-tRNAs during initial selection and accommodation, thereby reducing erroneous ejection of cognate aa-tRNAs during kinetic proofreading.

#### Stop Codon Decoding by eRF1

Unlike translation elongation, the factors and mechanisms mediating translation termination are not conserved between prokaryotes and eukaryotes (Dever and Green, 2012). This includes the mechanism of stop codon recognition, as well as the role of termination-associated GTPases. Recent cryo-EM structures have revealed how accommodated eRF1 interacts with stop codons (Brown et al., 2015b, Matheisl et al., 2015). However, the mechanism of stop codon recognition during the initial eRF1⋅eRF3 interaction with 80S ribosomes was unclear, as earlier structures had only visualized this complex at moderate resolution (Taylor et al., 2012, des Georges et al., 2014, Preis et al., 2014, Muhs et al., 2015). To address this problem, programmed RNCs with a UGA stop codon in the A site were used to isolate three intermediate states along the canonical termination pathway: (1) delivery of eRF1 to the stop codon by eRF3; (2) accommodated eRF1; and (3) accommodated eRF1 after ABCE1 recruitment (Figures 2A, S1, S2, and S4B–S4D) (Brown et al., 2015b).

The structures show that the stop codon maintains the same compacted geometry and interactions with the eRF1 N domain (Brown et al., 2015b, Matheisl et al., 2015) throughout the termination pathway (Figures 2B and S4B–S4D), despite large rearrangements of the M and C domains of eRF1 (see below). In this configuration, the +2 and +3 stop codon bases stack with a flipped-out A1825, and the base following the stop codon (+4) stacks with G626 in the anti-conformation (Figures 2B, 2C, and S4E) (Brown et al., 2015b, Matheisl et al., 2015). Improved density for the mRNA further reveals that the +5 base can stack with nucleotide C1698 of 18S rRNA, which protrudes into the mRNA channel (Figures 2B and 2C). The increased stability imparted by this additional stacking interaction explains why a +5 purine can increase the effectiveness of a “weak” stop codon with a +4 pyrimidine (McCaughan et al., 1995).

#### Recognition of Stalled Translation Complexes by Pelota

Pelota has been reported to bind stalled ribosomes with an empty A site as well as those with an mRNA-occupied A site without sequence preference (Shoemaker et al., 2010). To determine the basis for this sequence-independent engagement by the rescue complex, we utilized our reconstitution method to assemble 80S⋅Pelota⋅Hbs1l complexes with an A site that lacked mRNA (assembled on a truncated mRNA), or that contained either the UGA stop codon or the AAA sense codon (due to translation stalling within a poly(A) tail) (Figures 3A, S1, and S2). The complex assembled on a truncated mRNA shows that the β3′-β4′ loop of Pelota extends from the N domain to protrude into the empty mRNA channel, following the path normally taken by mRNA (Figures 3B and S4F). A similar path is taken by the shorter β3′-β4′ loop of yeast Dom34 as observed at moderate resolution (Becker et al., 2011). However, the higher-resolution information in our map allows the details of this interaction to be analyzed. The highly conserved residue (Arg45) at the top of the β3′-β4′ loop appears to play an anchoring role in the complex. Arg45 can hydrogen bond with His100, which is part of a conserved (Y/F/H)HT sequence on β6′ that interacts with 18S rRNA (Figure 3C). Arg45 is also part of a wider hydrogen-bonding network that includes the decoding nucleotide G626 in the anti-conformation (Figure 3C). Residues 60-61 prevent the decoding nucleotide A1824 from flipping out of h44, while A1825 is flipped out and interacts with Arg62. Together, these and other potential interactions with uS3 and uS5 probably stabilize the otherwise flexible and poorly conserved loop (Kobayashi et al., 2010). Thus, the β3′-β4′ loop is well positioned to sense A site occupancy.

Surprisingly, in both reconstructions containing mRNA sequence downstream of the P site, the conformation of the β3′-β4′ loop in the mRNA channel is unchanged, and we observe little to no density for the mRNA in the A site, while the mRNA upstream of the A site is also noticeably more disordered (Figures 3D and 3E). The high occupancy of Pelota⋅Hbs1l in these datasets (∼26%), the purity of our biochemically isolated complexes, and no evidence of endonucleolytic mRNA cleavage in our samples suggest that Pelota⋅Hbs1l is not recognizing a minor population of ribosomes that do not contain mRNA in the A site. Instead, we favor a mechanism by which the Pelota β3′-β4′ loop is able to bind a variety of mRNA substrates and, in doing so, destabilizes the mRNA within the channel. In support of this, the moderate-resolution structure of Dom34⋅Hbs1 bound to ribosomes stalled by mRNA secondary structure (Becker et al., 2011) also noted poor density within the mRNA channel.

#### Distinct Molecular Interactions Govern Decoding Factor Selection

Comparisons of the overall architectures (Figures 1A, 2A, and 3A) and the decoding centers of our structures (Figures 1B, 2B, and 3B) suggest that the mammalian ribosome does not display translational status-specific cues to favor engagement by a particular decoding factor⋅translational GTPase complex. Instead, successful recognition relies on decoding factors exploiting the inherent plasticity of the mRNA and the ribosomal decoding center, with sampling preference being biased by the overall abundance and local concentrations of each complex.

Highly specific interactions form between decoding factors and mRNA sequences during elongation and termination. In particular, the ribosomal protein eS30 may contribute to increasing the stringency of sense codon decoding in eukaryotes relative to bacteria. By contrast, the β3′-β4′ loop of Pelota invariably inserts into the mRNA channel and follows the path normally taken by mRNA, regardless of the mRNA substrate (Figure 3). Having to compete with mRNA for the channel may mean that Pelota⋅Hbs1l undergoes more futile attempts to engage the ribosome than other decoding complexes. This barrier and the relatively low abundance of Pelota and Hbs1l (Geiger et al., 2012) probably renders Pelota⋅Hbs1l a poor competitor for elongating or terminating ribosomes. Only during protracted periods of stalling, or with a truncated mRNA, would the likelihood of the β3′-β4′ loop engaging the ribosomal A site increase.

Once inserted, the loop maintains the mRNA in a less stable state that may facilitate subsequent endonucleolytic cleavage and/or ribosome splitting. Although endogenous substrates of Pelota⋅Hbs1l remain poorly characterized, this model is supported by in vitro studies showing that Pelota⋅Hbs1l is more effective at mediating the recycling of ribosomes stalled on mRNAs with shorter lengths extending 3′ of the P site (Pisareva et al., 2011, Shoemaker and Green, 2011) and suggests that ribosomes on more flexible mRNA (for example, mRNA that has already been cleaved) or that are not engaged in active translation are better substrates for Pelota⋅Hbs1l (van den Elzen et al., 2010, Guydosh and Green, 2014).

### Implications of Specialized GTPase Complexes for Eukaryotic Translation

#### Ribosomal Movements upon Decoding Complex Engagement

**Figure 4:**
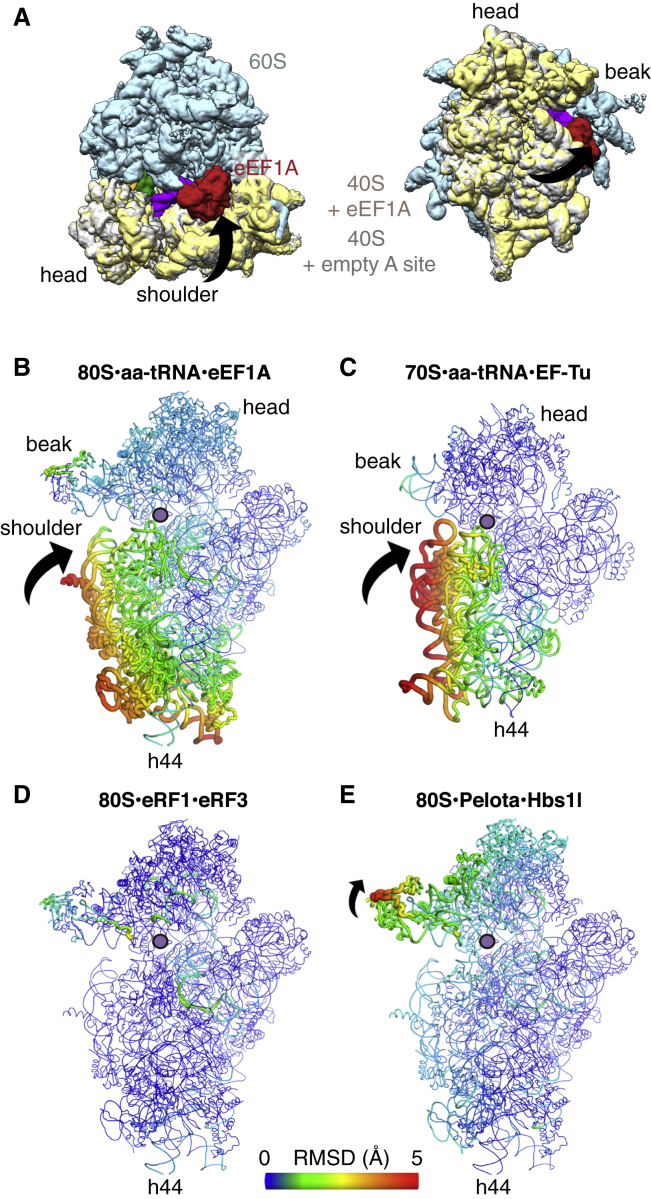
Conformational Responses of the Ribosome to Decoding Complexes (A) EM map of the elongation complex (colored) superposed on a ribosome with an empty A site (gray small subunit), demonstrating the movement corresponding to domain closure (illustrated by the arrow). The shoulder region of the small subunit moves toward the large subunit, which maximizes the contacts between a translational GTPase and the ribosome, particularly with the GTPase center. (B–E) Worm diagrams colored by pairwise root-mean-square deviation (RMSD) of the small subunits of (B) the elongation complex relative to a ribosome with an empty A site, (C) of a bacterial elongation complex (PDB: 5AFI) relative to an empty ribosome (PDB: 4UY8), and of the (D) termination and (E) rescue complexes relative to the same reference as in (B). The directions of movements are indicated by arrows. The A site is indicated with a purple dot.

Cognate codon-anticodon recognition in the decoding center of bacterial ribosomes induces a subtle but large-scale conformational change in the small subunit (SSU), referred to as domain closure (Ogle et al., 2001, Ogle et al., 2002) (Figure 4A). This movement has been proposed to induce a tighter fit around the codon-anticodon helix and to help activate the translational GTPase. To determine how the mammalian ribosome responds to recognition by different decoding factors, we compared each decoding complex to an unrotated rabbit ribosome containing a P-site peptidyl-tRNA and an unoccupied A site and GTPase-associated center.

In the mammalian elongation complex, there is a pronounced rotation of the shoulder of the SSU toward the intersubunit interface (Figures 4A and 4B) that resembles domain closure in bacteria (Ogle et al., 2001, Ogle et al., 2002). This movement raises the rRNA of the SSU platform by ∼3–4 Å to closely contact domain 2 of eEF1A. For an accurate comparison and to avoid the possible influences of crystal contacts, we re-analyzed the conformational changes that occur in bacteria using high-resolution cryo-EM structures of *E. coli* ribosomes with (Fischer et al., 2015) and without (Bischoff et al., 2014) an A-site tRNA (Figure 4C). This shows that domain closure in bacteria and mammals is broadly conserved, although the rotation of the shoulder around rRNA h44 is slightly more pronounced in the bacterial structure.

Domain closure appears to be a specific response to aa-tRNA selection and does not occur in the presence of either eRF1 or Pelota (Figures 4D and 4E). However, subtle conformational changes can be observed, particularly in the rescue complex where displacement of the head of the SSU (Figure 4E) may help the A site to accommodate the N domain of Pelota. The exclusivity of domain closure to elongation suggests that the precise positioning of elements within the decoding center is crucial for this large-scale movement. Only in the elongation complex are both decoding nucleotides A1824 and A1825 flipped out of h44 (Figures 1B, 2B, and 3B). This configuration may work with G626 and neighboring proteins, particularly uS12, to tether the interactions of the decoding center to propagate movement.

Consistent with this, our structures reveal considerable differences in the position of uS12 relative to the mRNA and decoding nucleotides in each complex (Figures S4A, S4E, and S4F). Direct interactions between uS12, the mRNA, and the flipped-out A1824 nucleotide occur only in the presence of a cognate aa-tRNA. In eukaryotes, mutations in uS12 influence translation fidelity (Alksne et al., 1993, Loenarz et al., 2014), similar to the *restrictive* mutations in bacteria (Ogle et al., 2002), supporting a role for uS12 in stabilizing the conformation induced by codon recognition. The same architecture may be induced with near-cognate tRNAs during crystallization (Demeshkina et al., 2012). However, we believe this suggests that non-cognate tRNAs have to go through the same activated state as cognate tRNAs in order to be selected, rather than implying that domain closure is not an intrinsic part of decoding. In physiological conditions, the probability of reaching the activated state is likely much more favored for cognate interactions than for non-cognate ones. Consistent with this, mutations expected to impede domain closure are associated with hyperaccurate phenotypes but a corresponding loss of translational efficiency (Andersson et al., 1986, Ogle and Ramakrishnan, 2005).

#### Pre-accommodation Decoding Factor⋅GTPase Interactions

**Figure S5:**
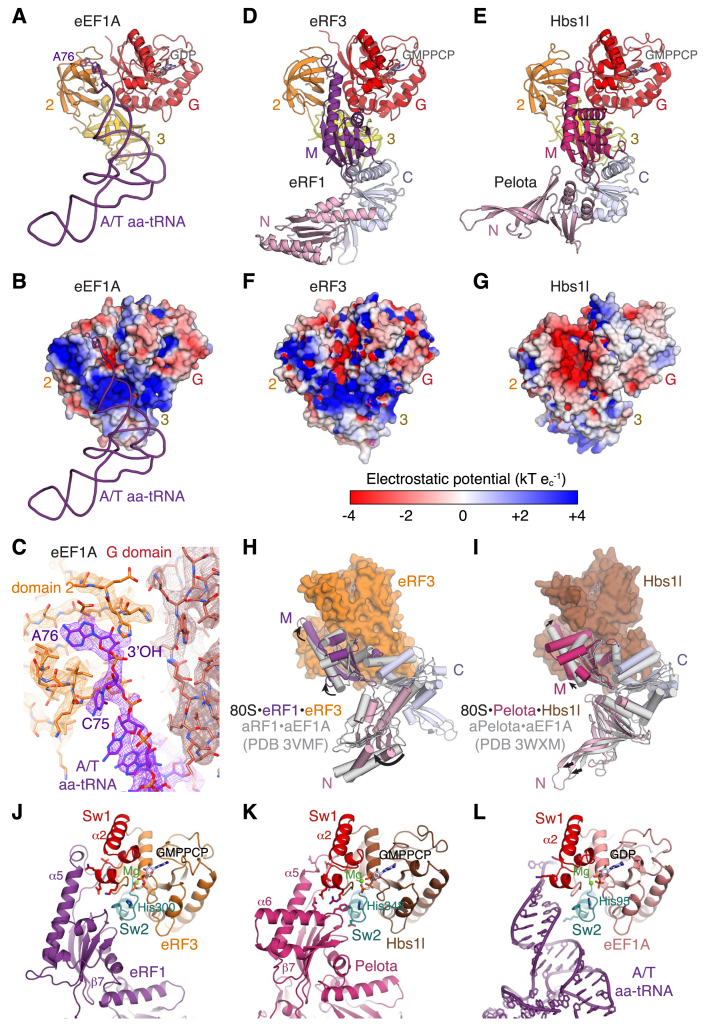
Details of Pre-accommodation Architectures, Related to Figure 5 (A) The acceptor stem of aa-tRNA (purple) binds in a cleft between the G domain (red) and domains 2 (orange) and 3 (yellow) of eEF1A. (B) Surface model of eEF1A colored by electrostatic potential (same view as panel A). (C) EM map density contoured at 7σ and models of the interactions between the 3′ end of aa-tRNA (purple) and domain 2 (orange) and G domain (red) of eEF1A. (D and E) The M domains of (D) eRF1 and (E) Pelota bind their respective GTPase partners in a cleft analogous to where aa-tRNA binds eEF1A. Structures are aligned as in panel (A). (F and G) Surface model colored by electrostatic potential of (F) eRF3, and (G) Hbs1l. (H and I) Superposition of (H) the crystal structure of aRF1⋅aEF1A⋅GTP (gray) on ribosome-bound eRF1⋅eRF3⋅GMPPCP or of (I) the crystal structure of aPelota⋅aEF1A⋅GTP (gray) on ribosome-bound Pelota⋅Hbs1l⋅GMPPCP via domains 2 and 3 of the GTPase. Upon ribosome binding, the N domain of the decoding factor is reoriented, while the M domain forms additional contacts with the G domain of the GTPase. (J and K) Interactions between the M domains of (J) eRF1 or of (K) Pelota with the G domain of the respective GTPase. The β7-α5 loop, which harbors the GGQ motif of eRF1, makes interactions with the Switch 1 (Sw1, red) motif, and additional interactions are formed with the Switch 2 (Sw2, teal) motif harboring the catalytic histidine. (L) The backbone and CCA end of A/T aa-tRNA also interacts with catalytically important motifs of the G domain of eEF1A.

The absence of domain closure in the termination and rescue complexes suggests that these decoding factors may directly communicate signals from the decoding center to the GTPase. Decoding factors bound to translational GTPases adopt a pre-accommodated conformation on the ribosome that prevents the decoding factor from engaging the PTC. For aa-tRNAs, this pre-accommodated state is referred to as the A/T state, which acts as a paradigm for understanding the role of this conformation during decoding. In the pre-accommodated state, the acceptor- and T-stems of the A/T aa-tRNA run parallel to, and interact with, the adjoined β-barrel domains of eEF1A at the interface with the G domain (Figures S5A and S5B), similar to recognition of aa-tRNAs by EF-Tu (Schmeing et al., 2009). Despite the aa-tRNA representing a mixture of species, the density for the 3′ CCA is well defined (Figure S5C). The aminoacylated terminal adenosine (A76) packs against the outside of the domain 2 β-barrel in a pocket formed by two protruding loops (β7-β8 and β10-β11; Figure S5C), while the aminoacyl group is oriented into a spacious cavity between domain 2 and the G domain that can accommodate all 20 amino acids.

The M domains of eRF1 and Pelota bind their respective translational GTPase in the same cleft between the G domain and domain 2 (Figures S5D and S5E) (Becker et al., 2011, Taylor et al., 2012). In both structures, the β7-α5 tip of the M domain (which harbors the catalytic GGQ motif in eRF1) follows the path of the 3′ CCA of the aa-tRNA but does not extend as far as the staggered pockets in eEF1A that bind A76 and the variable aminoacyl group. Although these pockets exist in eRF3 and Hbs1l, the lining residues are not conserved; indeed, the characteristics of the interface between each decoding factor and GTPase partner differ considerably (Figures S5B, S5F, and S5G). Compared to eEF1A, both eRF3 and Hbs1l contain a more electronegative cleft to bind the positively charged region around the β7-α5 tip, which is needed to interact with the phosphate backbone of rRNA in the PTC after accommodation. Thus, prior to GTP hydrolysis, high-affinity binding sites in translational GTPases maintain decoding factors in an unproductive conformation, in which the 3′ CCA of aa-tRNA or catalytic GGQ motif of eRF1 is held over 80 Å from the P-site tRNA ester bond in the PTC.

Comparison with the crystal structures of GTP-bound ternary complexes (Kobayashi et al., 2010, Kobayashi et al., 2012) reveals that the N domains of the decoding factors are oriented differently on the ribosome to engage the decoding center (Figures S5H and S5I). This may propagate conformational changes through the factor and establish additional interactions between the M domain and the GTPase, particularly with the G domain, which harbors three functionally important motifs (the P loop, and the switch 1 and switch 2 loops) that are thought to form productive contacts with the sarcin-ricin loop (SRL) of the ribosome to activate GTP hydrolysis (Voorhees and Ramakrishnan, 2013). In the termination complex, the β7-α5 loop of eRF1 contacts the end of the switch 1 loop of eRF3 (residues 279–281) (Figure S5J). Similarly, the equivalent region of Pelota interacts with the α3-α4 loop of the Hbs1l switch 1 region, while the conserved PGF motif in the β8-α6 loop (Lee et al., 2007) together with His244 of Pelota recognize part of the Hbs1l switch 2 loop (Figure S5K). Hence, these interactions may permit the decoding factor to directly facilitate the precise positioning of the GTPase G domain for productive GTP hydrolysis.

While direct communication via the decoding factor may be particularly important in the termination and rescue complexes where domain closure was not observed (Figure 4), the phosphate backbone of the acceptor arm of A/T aa-tRNA also makes potential electrostatic interactions with both switch regions of eEF1A (Figure S5L). This is consistent with observations that an intact aa-tRNA is necessary to trigger EF-Tu hydrolysis (Piepenburg et al., 2000), and that tRNA mutations can increase GTPase activation rates (Cochella and Green, 2005).

#### Didemnin B Prevents eEF1A Dissociation

**Figure 5:**
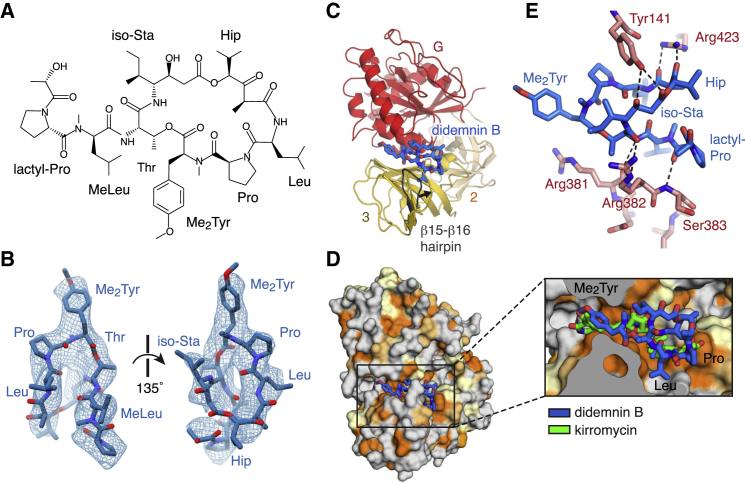
The Didemnin B Binding Site (A) Chemical structure of didemnin B. (B) Fit of the model of didemnin B (blue) to the EM map density contoured at 5σ. (C) Didemnin B binds at the interface between the G domain (red) and domain 3 (yellow) of eEF1A. Domain 2 is shown in orange. Relative to the eEF1A crystal structure (PDB: 4C0S; gray), the β15-β16 hairpin packs against didemnin B. (D) Didemnin B occupies a hydrophobic pocket of eEF1A (orientated as in C), which corresponds to the binding site for kirromycin (green) on EF-Tu. (E) Hydrogen-bonding interactions between didemnin B (blue) and eEF1A (pink). See also Figures S5 and S6.

**Figure S6:**
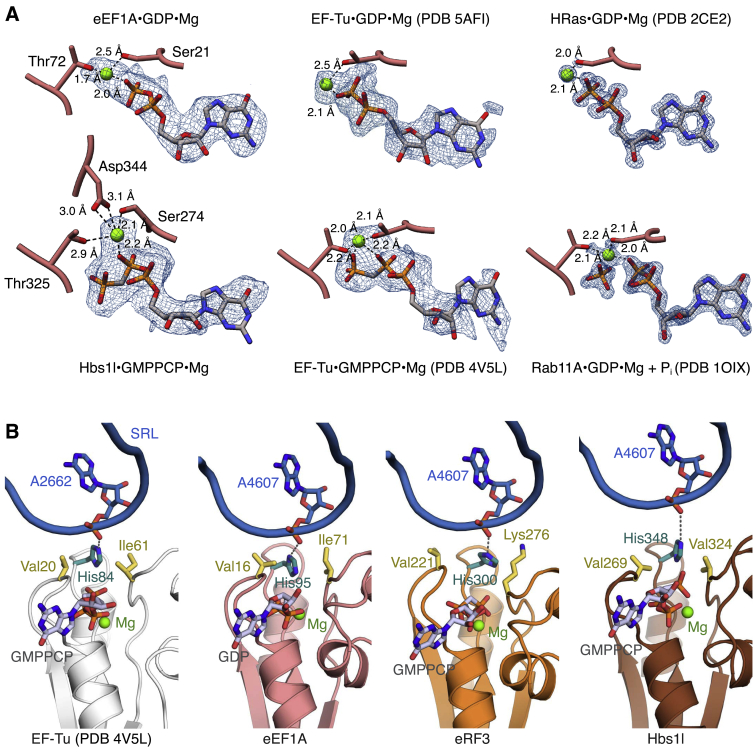
GTPase Active Sites, Related to Figure 5 (A) EM map density and model for GDP and GTP analogs in the indicated structures. eEF1A-bound GDP density is contoured at 7σ; Hbs1l-bound GMPPCP density is contoured at 6σ. Coordinating residues (pink) and magnesium ions (green) are indicated. (B) Interactions of the sarcin-ricin loop (SRL) with the catalytic histidine (teal) of the indicated GTPase. The residues of the hydrophobic gate are indicated in yellow.

The structure of the elongation complex shows that didemnin B, a naturally occurring branched cyclic depsipeptide protein synthesis inhibitor (Rinehart et al., 1981, Li et al., 1984) (Figures 5A and 5B), traps eEF1A in a post-hydrolysis GDP-bound state (Figure S6A) by occupying a cleft between the G domain and domain 3 of eEF1A that is ∼20 Å from the GTPase active site (Figure 5C).

Didemnin B binding appears to be predominantly stabilized by hydrophobic interactions, with the Leu and methylleucine (MeLeu) moieties occupying a hydrophobic pocket on the surface of the domain 3 β-barrel (Figure 5D). Didemnin B is further held in place by a solvent-exposed β-hairpin insertion (β15-β16, residues 375–391) of the domain 3 β-barrel, which is absent in bacterial EF-Tu. Compared to the crystal structure of rabbit eEF1A in its non-ribosome-bound state (Crepin et al., 2014), the tip of this hairpin is displaced by ∼4 Å to pinch didemnin B against the G domain (Figure 5C). The conserved loop residues 381–383 potentially form hydrogen bonds with the backbone of the branch and Thr moiety of didemnin B (Figure 5E) that may not occur with the shorter branches in didemnin A or C, possibly explaining the greater potency of didemnin B (Rinehart et al., 1981).

The didemnin B binding site partially overlaps with that of the linear polyketide kirromycin on EF-Tu, despite the different chemical structures of the two compounds (Schmeing et al., 2009) (Figure 5D). Based on this observation, we propose a conserved mechanism (Schmeing et al., 2009) that didemnin B serves to increase the effective number of contacts between the GDP-bound G domain and domain 3 to prevent the inter-domain rotation that is necessary for eEF1A to release aa-tRNA and dissociate from the ribosome. Recently, didemnin B and ansatrienin B have been shown to compete with ternatin for binding to eEF1A (Carelli et al., 2015), and mutations in eEF1A Ala399, adjacent to the β15-β16 hairpin, were found to confer decreased sensitivity to didemnin B, ternatin, and another structurally unrelated natural product, nannocystin A (Carelli et al., 2015, Krastel et al., 2015). This suggests that these chemically diverse natural products share similar mechanisms of activity.

**Figure 6:**
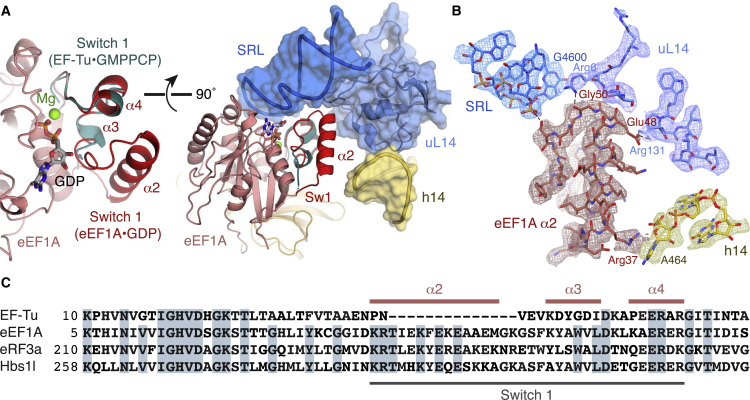
Interactions between the GTPase and the Ribosome (A) Comparison of the switch 1 loop (red) of eEF1A (pink) in the elongation complex with the EF-Tu switch 1 loop (teal) in the presence of GMPPCP (PDB: 4V5L) (left). The switch 1 (Sw1) loop interacts with proteins and rRNA from both the large (blue) and small (yellow) subunits of the ribosome (right). (B) EM map density and model of the interactions between the eEF1A switch 1 loop (red) with rRNA and proteins of the large (blue) and small (yellow) subunit. Density for rRNA is contoured at 9σ; density for eEF1A and uL14 is contoured at 5σ. (C) Sequence alignment of the switch 1 loop region of selected translational GTPases.

However, unlike bacterial elongation complexes trapped with kirromycin (Fischer et al., 2015, Schmeing et al., 2009), the switch 1 loop of eEF1A is ordered in the GDP-bound elongation complex (Figures 6A and 6B). This was surprising, as the switch 1 loop is thought to universally facilitate the gating function of GTPases by transitioning from an ordered to a disordered state upon GTP hydrolysis (Vetter and Wittinghofer, 2001, Voorhees and Ramakrishnan, 2013) and has so far only been observed in an ordered state in the presence of nonhydrolyzable GMPPCP (Voorhees et al., 2010). This suggests that disordering of the switch 1 loop in mammalian translational GTPases either occurs as an independent step not immediately linked to P_i_ release, or is stabilized as an indirect consequence of didemnin B binding.

#### Activated State of Eukaryotic Translational GTPases

Eukaryotic GTPases possess a short insertion (∼14 residues) relative to bacterial EF-Tu immediately preceding the switch 1 loop (Figure 6C). In our complexes, this insertion forms an amphipathic α helix (α2; Figures 6A–6C) connected by a short loop to a helical turn before adopting the same conformation observed in the EF-Tu⋅GMPPCP complex (Voorhees et al., 2010) (Figure 6A). In the elongation complex, the α2 helix lies across the surface of eEF1A to bury the hydrophobic face, while the polar residues on the other side interact with the ribosome. At the top of the α2 helix, Arg37 stacks with nucleotide A464 from h14 of SSU rRNA. The C-terminal part of the α2 helix and the following loop (residues 48–53) make multiple interactions with uL14: Glu48 of eEF1A potentially forms salt bridge with Arg131 of uL14, and contacts between the eEF1A loop with Arg6 and Gly7 of uL14 appear to stabilize the usually disordered N terminus of uL14. Additional contacts occur between Ser53 and nucleotide G4600 of the SRL (Figure 6B). A similar network of interactions is seen for the α2 helix of eRF3 and Hbs1l. Together, these eukaryotic-specific interactions may help to stabilize the switch 1 region, perhaps explaining why it is not disordered despite loss of the γ-phosphate in the didemnin B-stalled elongation complex.

An effect of the ordered switch 1 region in the elongation complex is that the eEF1A catalytic residues adopt the same conformation as seen in the “activated” state of EF-Tu trapped on the bacterial ribosome by GMPPCP (Figure S6B) (Voorhees et al., 2010). In this conformation, the eEF1A catalytic histidine His95 on the switch 2 loop is coordinated by the phosphate backbone of nucleotide A4607 of the SRL (His84 and A2662, respectively, in *E. coli*), and the hydrophobic gate formed by residues Val16 and Ile71 (Val20 and Ile61 in EF-Tu) appears to be in an open conformation. Similar configurations were observed in the termination and rescue complexes, which were reconstituted with GMPPCP (Figure S6B). Notably, the G domain of Hbs1l is further from the SRL, and the catalytic histidine (His348) is less strongly coordinated. This could increase the length of time Pelota⋅Hbs1l needs to be associated with the ribosome before hydrolysis occurs, thereby increasing the stringency for a productive encounter.

#### Specialization of Translational GTPases Regulates Initial Selection and Activation

Although the three translational GTPase partners share considerable structural similarity and superpose with root-mean-square deviation (RMSD) values between 1.4 and 1.9 Å, they cannot complement each other (Wallrapp et al., 1998) and possess divergent interfaces specialized to interact with their respective decoding factor (Figures S5B, S5F, and S5G). Similar sub-functionalization has not occurred in archaea, where aEF1α plays an omnipotent role to deliver aa-tRNA, aRF1, and aPelota to ribosomes (Saito et al., 2010).

Our structures suggest several advantages of having a dedicated translational GTPase for each decoding factor in maintaining overall translational fidelity. First, improved affinity between decoding factors and individual GTPases (Figures S5A–S5G), combined with distinct temporal and spatial distribution patterns, probably contribute to higher selectivity during decoding. Second, non-redundant pairing may allow for distinct mechanisms for communicating decoding events to the GTPase (e.g., Figure 4), possibly via direct interactions between the decoding factor and motifs needed for GTP hydrolysis (Figures S5J and S5L). Finally, specialized complexes may have different dissociation constants and basal activation barriers to GTP hydrolysis that could alter the general competitiveness of each decoding complex (Figures 4 and S6B).

### Conformational Changes Coordinate Decoding Factor Accommodation

**Figure 7:**
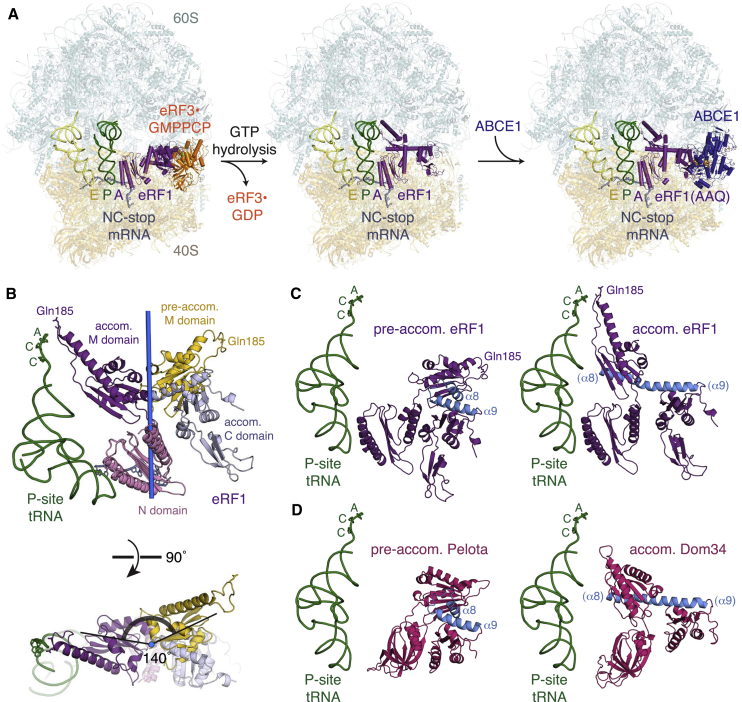
Conformational Changes during Accommodation (A) Structures of ribosomal complexes representing intermediates along the eukaryotic translation termination pathway. (B) The accommodated M domain (purple) of eRF1 is rotated by 140° relative to the pre-accommodated state (yellow). Gln185 of the catalytic GGQ motif, P-site tRNA (green), the N domains in both states (pink), the C domain (pale blue) in the accommodated state, and the axis of M domain rotation (blue) are shown. (C) Comparison of eRF1 (purple) in a pre-accommodated state (left) with an accommodated (right) conformation, showing straightening of α8 and α9 (blue) into a continuous helix upon accommodation. (D) Comparison of Pelota (pink) in a pre-accommodated state (left) with Dom34 (pink) in an accommodated (right) state (right; PDB: 3IZQ), revealing straightening of α8 and α9 (blue). See also Figure S7.

After GTP hydrolysis and GTPase dissociation, the decoding factor needs to accommodate fully into the PTC without dissociating from the ribosome. Our structural snapshots of the translation termination pathway reveal the conformational effects of accommodation on eRF1 and the ribosome (Figure 7A). After eRF3 dissociates, the M and C domains of eRF1 undergo large interdependent rotations relative to the static N domain. The pre-accommodated and accommodated M domains are related by a 140° rotation around Asp142 in the linker between the N and M domains (Figure 7B).

However, the driving force for this rearrangement may derive from the hinge (centered on residue 276) between helices α8 and α9 connecting the M and C domains, which are held at an acute kink (∼70°) by eRF3. Accommodation relieves this conformational strain by allowing α8 and α9 to straighten into a continuous α helix (Figure 7C). Comparing pre-accommodated Pelota with accommodated Dom34 (Becker et al., 2012) reveals a similar transition (Figure 7D). The confined environment around the decoding factors suggests that, as demonstrated for aa-tRNAs (Whitford et al., 2010), the accommodation pathway of eRF1 and Pelota is likely complex, comprising multiple steps. This may slow the rate of accommodation and provide more opportunities for the decoding factor to dissociate when interactions at the decoding center are suboptimal.

**Figure S7:**
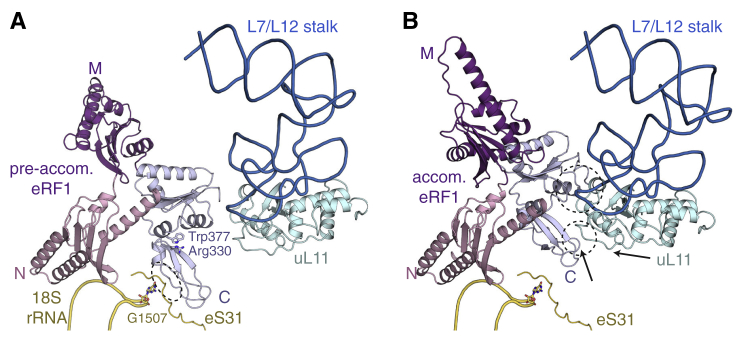
Conformational Changes during Decoding Factor Accommodation, Related to Figure 7 (A) The minidomain of pre-accommodated eRF1 (colored by domains) forms an interaction (circled) with eS31 (yellow) that is stabilized by G1507 of 18S rRNA. (B) Upon accommodation, the M (purple) and C (pale blue) domains of eRF1, and the L7/L12 rRNA stalk base (blue) supporting uL11 (light cyan) undergo conformational changes to establish new interactions (circled) between the eRF1 minidomain with uL11 and the L7/L12 stalk base. Arrows indicate the direction and magnitude of movement of the minidomain and uL11 from the pre-accommodated state.

A key structural difference between Pelota and eRF1 is a “minidomain” insertion in the eRF1 C domain (residues 328–373) (Figure S3B). The minidomain adopts different orientations during the termination pathway, although its movement is restricted by a stacking interaction between Arg330 at the top of the minidomain and Trp377 of the C domain (Figure S7A). In the recognition complex, the minidomain interacts with the N terminus of eS31 that wraps around the flipped-out G1508 nucleotide of SSU rRNA, which may facilitate initial binding of the ternary complex to the ribosome. During accommodation, the minidomain switches subunit partners: the contacts with eS31 are disrupted and new contacts with uL11 form, primarily via an interaction with the C-terminal tail of eRF1 (Figure S7B). Together, these may stabilize both the eRF1 C domain and the L7/L12 stalk base to facilitate ABCE1 binding (Brown et al., 2015b), which displaces the minidomain by another ∼1.5 Å and further stabilizes the interactions with uL11.

### Conclusions

Collectively, our structures suggest that specialization of eukaryotic decoding factor⋅translational GTPase complexes enhances overall translation fidelity and efficiency by allowing for distinct mechanisms of decoding (Figures 1, 2, and 3), activation (Figure 4), and accommodation (Figure 7). Our results also highlight fundamental differences from the bacterial system, including eukaryotic-specific elements that increase the stringency of sense codon decoding (Figure 1) and the absence of domain closure in certain decoding complexes (Figure 4). This implicates novel mechanisms for communicating information from the decoding center to eukaryotic translational GTPases, and subtle but important variations in the rates of GTPase activation and accommodation of eukaryotic decoding complexes. Together, these distinctions likely translate into decisive differences in the competitive advantage of each decoding complex for different ribosome-mRNA substrates.

## STAR★Methods

### Key Resources Table

**Table:** 

REAGENT or RESOURCE	SOURCE	IDENTIFIER
**Antibodies**		

Rabbit monoclonal anti-eEF1A1 antibody	Abcam	Cat. #ab140632
Rabbit polyclonal anti-uL6 antibody	Santa Cruz	Cat. #102085; RRID: AB_2182219
Rabbit polyclonal anti-uS9 antibody	Santa Cruz	Cat. #sc-102087; RRID: AB_2269633

**Chemicals, Peptides, and Recombinant Proteins**		

3XFlag-TEV WT Hbs1l (human)	Shao et al., 2013	N/A
3XFlag-TEV H348A Hbs1l-DN (human)	Shao et al., 2013	N/A
3XFlag-TEV eRF3a (human)	This study	N/A
WT eRF1 (human)	This study	N/A
eRF1(AAQ) (human)	Brown et al., 2015b	N/A
His-Pelota (human)	Shao et al., 2013	N/A
*E. coli* Poly(A) polymerase	New England Biolabs	Cat. #M0276L
3X Flag peptide	Sigma-Aldrich	Cat. #F4799
Anti-Flag M2 affinity resin	Sigma-Aldrich	Cat. #A2220
Ni-NTA agarose	QIAGEN	Cat. #30210
Didemnin B	This study (Jack Taunton)	CAS #77327-05-0
Cycloheximide	Sigma-Aldrich	Cat. #C4859; CAS #66-81-9
Emetine	Calbiochem	Cat. #324693; CAS #316-42-7
Anisomycin	Sigma-Aldrich	Cat. #A9789; CAS #22862-76-6
EasyTag L-[^35^S]-Methionine	Perkin Elmer	Cat. #NEG709A005MC
CAP (diguanosine triphosphate cap)	New England Biolabs	Cat. #S1404L
RNasin	Promega	Cat. #N251
SP6 polymerase	New England Biolabs	Cat. #M0207L
Creatine kinase	Roche	Cat. #127566
Creatine phosphate	Roche	Cat. #621714
Amino acid kit	Sigma	Cat. #09416

**Deposited Data**		

80S⋅empty A site density map	This study	EMDB: 4129
80S⋅aa-tRNA⋅eEF1A density map	This study	EMDB: 4130
80S⋅eRF1⋅eRF3 density map	This study	EMDB: 4131
80S⋅eRF1 density map	This study	EMDB: 4132
80S⋅eRF1⋅ABCE1 (combined) density map	This study	EMDB: 4133
80S⋅Pelota⋅Hbs1l (truncated mRNA) density map	This study	EMDB: 4134
80S⋅Pelota⋅Hbs1l (stop mRNA) density map	This study	EMDB: 4135
80S⋅Pelota⋅Hbs1l (polyA mRNA) density map	This study	EMDB: 4136
80S⋅Pelota⋅Hbs1l (combined) density map	This study	EMDB: 4137
80S⋅aa-tRNA⋅eEF1A atomic model	This study	PDB: 5LZS
80S⋅eRF1⋅eRF3 atomic model	This study	PDB: 5LZT
80S⋅eRF1 atomic model	This study	PDB: 5LZU
80S⋅eRF1⋅ABCE1 (combined) atomic model	This study	PDB: 5LZV
80S⋅Pelota⋅Hbs1l (truncated mRNA) atomic model	This study	PDB: 5LZW
80S⋅Pelota⋅Hbs1l (stop mRNA) atomic model	This study	PDB: 5LZX
80S⋅Pelota⋅Hbs1l (polyA mRNA) atomic model	This study	PDB: 5LZY
80S⋅Pelota⋅Hbs1l (combined) atomic model	This study	PDB: 5LZZ

**Experimental Models: Cell Lines**		

HEK293T	ATCC	CRL-3216

**Experimental Models: Organisms/Strains**		

*E. coli* BL21 (DE3)	Thermo Fisher	C600003
*E. coli* BL21 (DE3) pLysS	Thermo Fisher	C606003

**Recombinant DNA**		

pcDNA 3XFlag-TEV WT Hbs1l	Shao et al., 2013	N/A
pcDNA 3XFlag-TEV H348A Hbs1l	Shao et al., 2013	N/A
pcDNA 3XFlag-TEV eRF3a	This study	N/A
pRSETA 6XHis-TEV eRF1	This study	N/A
pRSETA 6XHis-TEV eRF1(AAQ)	Brown et al., 2015b	N/A
pSP64 3XFlag VHP Sec61-UGA(68)	Brown et al., 2015b	N/A
pSP64 3XFlag VHP Sec61-68	Shao et al., 2013	N/A
pSP64 3XFlag KRas	This study	N/A
Primer: SP64 5′ Fwd: TCATACACATACGATTTAGG	Sharma et al., 2010	N/A
Primer: SP64 Rev: CAATACGCAAACCGCCTC	Sharma et al., 2010	N/A
Primer: Val68 Rev: AACTTTGAGCCCAGGTGAATC	Shao et al., 2013	N/A

**Software and Algorithms**		

EPU software	FEI	https://www.fei.com/software/epu/
Motioncorr	Li et al., 2013	http://cryoem.ucsf.edu/software/driftcorr.html
Gctf v0.5	Zhang, 2016	http://www.mrc-lmb.cam.ac.uk/kzhang/Gctf/
RELION v1.4	Scheres, 2015	http://www2.mrc-lmb.cam.ac.uk/relion
ResMap v1.1.4	Kucukelbir et al., 2014	http://resmap.sourceforge.net/
Coot v0.8	Emsley et al., 2010	http://www2.mrc-lmb.cam.ac.uk/personal/pemsley/coot/
REFMAC v5.8	Murshudov et al., 2011	https://www2.mrc-lmb.cam.ac.uk/groups/murshudov/content/refmac/refmac.html
MolProbity v4.3	Chen et al., 2010	http://molprobity.biochem.duke.edu/
Phenix.elbow dev-2499	Adams et al., 2010	http://www.phenix-online.org/documentation/reference/elbow.html
UCSF Chimera v1.10.2	Pettersen et al., 2004	https://www.cgl.ucsf.edu/chimera/
PyMOL v1.7	Schrödinger, LLC	http://www.pymol.org

**Other**		

RRL in vitro translation mix	Sharma et al., 2010	N/A
TransIT 293	Mirus	MIR 2705

### Contact for Reagent and Resource Sharing

Requests for reagents may be directed to Lead Contact Ramanujan S. Hegde (rhegde@mrc-lmb.cam.ac.uk).

### Experimental Model and Subject Details

#### Cell Lines

HEK293T cells used for protein expression were maintained in DMEM (high glucose, GlutaMAX, pyruvate) with 10% fetal bovine serum.

### Method Details

#### Constructs

An SP64-based plasmid encoding 3X Flag-tagged Sec61β containing the autonomously folding villin headpiece (VHP) domain was used to generate transcripts truncated after the Val68 codon of Sec61β (Shao et al., 2013). For termination complexes, the same construct was modified to include the UGA stop codon after the Val68 codon (Brown et al., 2015b). To generate elongation complexes, the open reading frame of KRas was cloned after a 3X Flag tag in an SP64-based plasmid using conventional techniques. In vitro transcription reactions were performed using PCR products generated with primers that amplify from the SP6 promoter to either the 3′ UTR of the SP64 vector (Sharma et al., 2010) or to directly after Val68 of Sec61β (Shao et al., 2013).

The open reading frame of wild-type eRF1 (Brown et al., 2015b) was inserted after a N-terminal 6X His tag and a TEV cleavage site, and the Pelota open reading frame (Shao et al., 2013) was inserted before a C-terminal TEV cleavage site and 6X His tag in the pRSETA vector using conventional techniques. Point mutations in eRF1 were generated using Phusion mutagenesis (Brown et al., 2015b). The open reading frames of human Hbs1l (Shao et al., 2013) and eRF3a (Origene) were cloned after a 3X Flag tag in a pcDNA3-based vector using conventional procedures.

#### Purification of recombinant proteins

Wild-type and mutant eRF1 (eRF1(AAQ)) were expressed in, and purified from, *Escherichia coli* BL21(DE3) cells (Brown et al., 2015b). His-tagged Pelota was expressed and purified from *Escherichia coli* BL21(DE3) pLysS cells (Shao et al., 2013). Transformed cells were induced at A_600_ = 0.4-0.6 with 0.2 mM IPTG for 2 hr at 37°C and lysed with a microfluidizer in lysis buffer (1X PBS, pH 7.5, 250 mM NaCl, 10 mM imidazole, 1 mM DTT) containing 1X protease inhibitor cocktail (Roche). Lysates were clarified by centrifugation and the supernatant passed over a NiNTA column. After washing with 25 column volumes of lysis buffer, elutions were carried out with 250 mM imidazole in lysis buffer. Peak fractions were pooled, dialyzed overnight against 50 mM HEPES, pH 7.4, 150 mM KOAc, 5 mM Mg(OAc)_2_, 10 mM imidazole, 10% glycerol, 1 mM DTT. TEV protease was included during dialysis of eRF1 proteins. TEV protease and cleaved His tag were removed by passage over a NiNTA column.

Flag-tagged recombinant eRF3a and Hbs1l were purified from HEK293T cells (Shao et al., 2013). eRF3a was used for structural analysis as it is the primary release factor isoform used to terminate translation in mammalian cells, with eRF3b expression restricted to the brain (Chauvin et al., 2005). Transfection was with Mirus TransIT according to the manufacturer’s instructions. Cells were harvested after 3 days and lysed in 50 mM HEPES, pH 7.4, 100 mM KOAc, 5 mM Mg(OAc)_2_, 1% Triton X-100, 1 mM DTT, and 1X protease inhibitor cocktail (Roche). The post-nuclear supernatant lysate was incubated with anti-Flag (M2) agarose beads (Sigma) at 4°C for 1-1.5 hr. The resin was washed with 6 mL lysis buffer, followed by 6 mL 50 mM HEPES, pH 7.4, 250 mM KOAc, 5 mM Mg(OAc)_2,_ 1% Triton X-100, 1 mM DTT, followed by 6 mL elution buffer (50 mM HEPES, pH 7.4, 100 mM KOAc, 5 mM Mg(OAc)_2_, 1 mM DTT). Elution was carried out with two sequential incubations of one column volume of 0.1 mg/mL 3X Flag peptide (Sigma) in elution buffer for 25 min each at room temperature. The elutions were combined, flash frozen, and directly used for downstream assays.

#### In vitro transcription and translation reactions

Transcription reactions were conducted with ∼5-20 ng/μl purified PCR product, in 40 mM HEPES pH 7.4, 6 mM MgCl_2_, 20 mM spermidine (Sigma), 10 mM DTT, 0.5 mM ATP, 0.5 mM UTP, 0.5 mM CTP, 0.1 mM GTP (Roche), 0.5 mM CAP (NEB), 0.4-0.8 U/μL rRNasin (Promega), and 0.4 U/μL SP6 polymerase (NEB) at 37°C for 60 min (Sharma et al., 2010). In vitro translation reactions in a homemade rabbit reticulocyte (RRL) system containing 1/20 volume of transcription reaction, 0.5 μCi/μL ^35^S-methionine (Perkin Elmer EasyTag), nuclease-treated crude rabbit reticulocyte (Green Hectares), 20 mM HEPES, 10 mM KOH, 40 μg/mL creatine kinase (Roche), 20 μg/mL pig liver tRNA, 12 mM creatine phosphate (Roche), 1 mM ATP (Roche), 1 mM GTP (Roche), 50 mM KOAc, 2 mM MgCl_2_, 1 mM glutathione, 0.3 mM spermidine, and 40 μM of each amino acid except for methionine (Sigma), were at 32°C for 25 min unless otherwise indicated (Shao et al., 2013, Sharma et al., 2010).

#### Sample Preparations

##### Elongation complex

A transcript encoding 3X Flag-tagged KRas was translated in vitro. A final concentration of 50 μM didemnin B was added after 7 min to stall ribosome-nascent chain complexes (RNCs) at the stage of tRNA delivery by eEF1A and the reaction allowed to proceed to 25 min. 4 mL translation reaction was directly incubated with 100 μL (packed volume) of anti-Flag M2 beads (Sigma) for 1 hr at 4°C with gentle mixing. The beads were washed sequentially with 6 mL 50 mM HEPES, pH 7.4, 100 mM KOAc, 5 mM Mg(OAc)_2_, 0.1% Triton X-100, 1 mM DTT; 6 mL 50 mM HEPES, pH 7.4, 250 mM KOAc, 5 mM Mg(OAc)_2_, 0.5% Triton X-100, 1 mM DTT; and 6 mL RNC buffer (50 mM HEPES, pH 7.4, 100 mM KOAc, 5 mM Mg(OAc)_2_, 1 mM DTT). Two sequential elutions were carried out with 100 μL 0.1 mg/mL 3X Flag peptide (Sigma) in RNC buffer at room temperature for 25 min. The elutions were combined and centrifuged at 100,000 rpm at 4°C for 40 min in a TLA120.2 rotor (Beckman Coulter) before resuspension of the ribosomal pellet in RNC buffer containing 5 μM didemnin B. The resuspended RNCs were adjusted to 120 nM and directly frozen to grids for cryo-EM analysis.

##### Termination complexes

3X Flag-tagged Sec61β containing the autonomously-folding villin headpiece domain with a UGA stop codon was translated in vitro with 0.5 μM eRF1(AAQ) to trap termination complexes (Brown et al., 2015b). After 25 min, translation reactions were adjusted to 750 mM KOAc, 15 mM Mg(OAc)_2_ and spun on a 0.5M sucrose cushion containing 50 mM HEPES, pH 7.4, 750 mM KOAc, 15 mM Mg(OAc)_2_ at 100,000 rpm for 1 hr at 4°C in a TLA100.3 rotor (Beckman Coulter). The ribosome pellets from 4 mL translation reactions were resuspended in RNC buffer and incubated with 100 μL (packed volume) of anti-Flag M2 beads (Sigma) for 1-1.5 hr at 4°C with gentle mixing. The beads were washed sequentially with 6 mL 50 mM HEPES, pH 7.4, 100 mM KOAc, 5 mM Mg(OAc)_2_, 0.1% Triton X-100, 1 mM DTT; 6 mL 50 mM HEPES, pH 7.4, 250 mM KOAc, 5 mM Mg(OAc)_2_, 0.5% Triton X-100, 1 mM DTT; and 6 mL RNC buffer. Two sequential elutions were carried out with 100 μL 0.1 mg/mL 3X Flag peptide (Sigma) in RNC buffer at room temperature for 25 min. The elutions were combined and incubated with wild-type eRF1, wild-type eRF3, and 0.5 mM GMPPCP to generate the eRF1-eRF3 complex, or with wild-type eRF1, wild-type eRF3, and 0.5 mM GTP to generate the accommodated eRF1 complex. The reactions were centrifuged at 100,000 rpm at 4°C for 40 min in a TLA120.2 rotor (Beckman Coulter) before resuspension of the ribosomal pellet in RNC buffer containing ∼600 nM of recombinant eRF1 and eRF3 with 1 μM GMPPCP or GTP. Complexes containing eRF1(AAQ) and ABCE1 were prepared as previously (Brown et al., 2015b).

##### Rescue complexes

3X Flag-tagged Sec61β containing the autonomously-folding villin headpiece domain truncated after Val68 of Sec61β without or with a polyA tail was translated in vitro as previously described (Shao et al., 2013). After 7 min, an excess of dominant negative Hbs1l was added and the translation reaction allowed to proceed to 25 min before being isolated through a high salt cushion and affinity purified via the Flag-tagged as described above. The combined elutions in RNC buffer were incubated with Pelota, wild-type Hbs1l, and 0.5 mM GMPPCP to assemble stall-recognition complexes. The reactions were then centrifuged at 100,000 rpm at 4°C for 40 min in a TLA120.2 rotor (Beckman Coulter) before resuspension of the ribosomal pellet in RNC buffer containing ∼600 nM of recombinant Pelota and WT Hbs1l with 1 μM GMPPCP. The same strategy was used to assemble the rescue complex on a stop codon-containing substrate, except that the substrate contained a UGA stop codon after Val68 and was translated in the presence of eRF1(AAQ).

##### Reference table for the biological composition of final complexes used for cryo-EM

**Table:** 

Complex	**Ribosome (∼120 nM)**	**mRNA substrate (see**Figure S1**)**	**Recombinant proteins (∼600 nM each)**	**Other**
**80S⋅aa-tRNA⋅eEF1A**	Rabbit (RRL)	Long NC	None	5 μM didemnin B
**80S⋅eRF1⋅eRF3**	Rabbit (RRL)	NC-stop (UGA)	Human WT eRF1	1 μM GMPPCP
			Human WT eRF3	
**80S⋅Pelota⋅Hbs1l**	Rabbit (RRL)	Trunc. NC OR	Human WT Pelota	1 μM GMPPCP
		polyA NC OR	Human WT Hbs1l	
		NC-stop (UGA)		
**80S⋅eRF1**	Rabbit (RRL)	NC-stop (UGA)	Human WT eRF1	1 μM GTP
			Human WT eRF3	
**80S⋅eRF1⋅ABCE1 (****Brown et al., 2015b****)**	Rabbit (RRL)	NC-stop	eRF1(AAQ)	None

#### Cryo-EM grid formation

R2/2 cryo-EM grids (Quantifoil) were covered with continuous carbon (estimated to be 50 Å thick) and glow discharged to increase hydrophilicity. The grids were transferred to a Vitrobot MKIII (FEI) with the chamber set at 4°C and 100% ambient humidity. Aliquots of purified RNCs (3 μL, ∼120 nM concentration in 50 mM HEPES pH 7.4, 100 mM KOAc, 5 mM Mg(OAc)_2_, 1 mM DTT plus any additions as detailed in the Reference table above) were applied to the grid and incubated for 30 s, before blotting for 3 s to remove excess solution, and vitrified in liquid ethane.

#### Miscellaneous biochemistry

SDS-PAGE was with 10% or 12% Tris-tricine polyacrylamide gels run at 100 V for 85-90 min. For autoradiography and direct visualization of protein bands, gels were fixed and stained with Coomassie R250, destained and directly imaged, or dried and exposed on MR film (Kodak Carestream BioMax). For immunoblotting, gels were transferred to 0.2 μm nitrocellulose membrane (Bio-Rad) in a wet transfer system at 100V for 50 min. Blots were blocked and incubated with primary and secondary antibodies in 5% milk in PBS + 0.1% Tween. Antibodies were used at the following concentrations: 1:4000 αHbs1l, 1:4000 αABCE1, 1:1000 αeRF1, 1:1000 αeEF1A, 1:100 αuL6, and 1:100 αuS9. Secondary antibodies were used at 1:2500 or 1:5000.

Functional assays were conducted with ^35^S-methionine-labeled RNCs isolated under high salt conditions and affinity purified via the Flag tag exactly as described for cryo-EM grid preparation. The radiolabeled RNCs were then incubated with the recombinant proteins, 1 mM puromycin, or 0.5 mM GTP or GMPPCP at 32°C for 15 min before analysis by SDS-PAGE and autoradiography.

To sequence 28S rRNA, ribosomes were isolated from crude RRL from two rabbits under high salt conditions, and the RNA extracted using the RNeasy system (QIAGEN). Electrophoresis on 5% TBE-acrylamide gels and toluidine blue staining verified high recovery of 28S and 18S rRNA bands. The RNA sample was reverse transcribed with ArrayScript reverse transcriptase (Thermo Fisher) according to the manufacturer’s instructions and used for PCR reactions to amplify and sequence portions of the 28S sequence with Sanger sequencing. This revealed some rabbit-to-rabbit variability, and allowed for certain portions (but not all) of the 28S rRNA sequence to be determined with high confidence based on alignments with highly conserved regions. These regions were incorporated into the final model (see below).

#### Data collection

Details of the data collection for each complex are presented in Table S1. All micrographs were taken using quasi-automated data collection (EPU software, FEI) on a Titan Krios microscope equipped with a XFEG electron source using 300 kV acceleration voltage. Images were recorded on a Falcon II direct electron detector (FEI). For the termination and rescue complexes, a dose rate of ∼30 electrons per Å^2^ per second was used at a calibrated magnification of 104,478, resulting in a pixel size of 1.34 Å. Movie frames were collected at a rate of 16 s^-1^, with total exposures of 1.0-1.1 s. For the elongation complex and the comparative complex with an empty A site (Figure S2), a higher magnification (134,615, resulting in a pixel size of 1.04 Å) and a higher dose rate (∼40 electrons per Å^2^ per second) were used. In total, 13 independent data collections were used to collect 17,681 micrographs, from which nine structures were solved at resolutions ranging from 3.1 to 4.0 Å.

#### Image Processing

Details for the processing of each complex are presented in Table S1. Movies frames were aligned using whole-image motion correction (Li et al., 2013) to reduce beam-induced blurring of the images. Micrographs that displayed evidence of astigmatism, charging, contamination, and poor contrast were excluded. Parameters of the contrast transfer function for each motion-corrected micrograph were obtained using Gctf (Zhang, 2016). Ribosome particles were selected from the images using the interactive semi-automatic swarm tool in the e2boxer.py program of EMAN2 (Tang et al., 2007) or with semi-automated particle picking implemented in RELION 1.4 (Scheres, 2015). Reference-free two-dimensional class averaging was used to discard non-ribosomal particles, with those picked using RELION subjected to an additional sorting step (Scheres, 2015).

Particles retained after two-dimensional classification underwent an initial three-dimensional refinement using a 30 Å low-pass filtered cryo-EM reconstruction of a rabbit ribosome (EMDB 3039) as an initial model. After refinement, statistical particle-based movie correction was performed in RELION 1.4 (Scheres, 2015) that included a resolution and dose-dependent model for the radiation damage, in which each frame is B-factor weighted as estimated from single-frame reconstructions (Scheres, 2014).

The resulting ‘shiny’ particles were then subjected to three-dimensional classification to separate different compositions and conformations of the ribosome complexes and isolate particles with high occupancy of the desired factors. This step was omitted for the 80S⋅aa-tRNA⋅eEF1A complex. Particles retained after three-dimensional classification were subjected to focused classification with signal subtraction (FCwSS) (Bai et al., 2015) to further isolate particles containing the desired factor. After FCwSS, an additional round of 3D classification and refinement were used to obtain the final maps.

Reported resolutions are based on the Fourier shell correlation (FSC) 0.143 criterion (Rosenthal and Henderson, 2003). High-resolution noise substitution was used to correct for the effects of a soft mask on FSC curves (Chen et al., 2013). Before visualization, density maps were corrected for the modulation transfer function of the Falcon II detector and then sharpened by applying a negative B-factor that was estimated using automated procedures (Rosenthal and Henderson, 2003). Local resolution was quantified using ResMap (Kucukelbir et al., 2014).

#### Model building

##### Ribosome

Both subunits of the mammalian ribosome (PDB accession code 3JAH) (Brown et al., 2015b) were individually docked into the map with Chimera (Pettersen et al., 2004). The atomic models of the ribosomal proteins and 18S rRNA were modified in Coot v0.8 to agree with the rabbit sequences and optimized for fit to density using rigid body fitting followed by real-space refinement in Coot (Brown et al., 2015a, Emsley et al., 2010). Where possible, the atomic model of 28S rRNA was modified to reflect the rabbit sequence (OryCun2.0 GCA_000003625.1). However, since this sequence had insufficient coverage, we also attempted to sequence the 28S rRNA directly from ribosomes extracted from RRL (see above for experimental procedures). The model was then modified to agree with regions with high sequencing confidence (bases 725-965, 1271-2888, 3584-3867) or, in well-conserved areas, to better match the complete 28S rRNA sequences from human (NCBI accession NR_003287.2) and rat (a closely related rodent; NCBI accession NR_046246.1). Human numbering is used for the rRNA (NCBI accession NR_003287.2 for 28S, and X03205.1 for 18S). See Table S3 for the numbering and sequence in the ribosome model aligned with the human reference.

##### Elongation complex

Because our structure represents a mixture of species, the starting models for the P- and E-site tRNAs and the mRNA were taken from our previous structure (PDB accession code 3JAH) (Brown et al., 2015b). P-site tRNA^Val^ was also used as an initial model for the A-site tRNA. The fit of the tRNAs and mRNA to the density were optimized using rigid body fitting and real space refinement. The crystal structure of yeast eEF1A (PDB accession code 1F60) (Andersen et al., 2000) was docked into density at the GAC. The switch I loop region was taken from the structure of EF-Tu bound to GMPPNP (PDB accession code 2C78) (Parmeggiani et al., 2006). The model of eEF1A was modified to the rabbit sequence (UniProt ID: P68105) and manually fit to density.

The small molecule crystal structure of didemnin B (Hossain et al., 1988) was docked into empty density near eEF1A and adjusted in Coot using real space refinement with chemical restraints generated using Phenix.elbow (Adams et al., 2010). The geometry of didemnin B model was analyzed using Mogul, a molecular-geometry library derived from the Cambridge Structural Database (CSD) (Bruno et al., 2004). Some of the restraints generated from Phenix.elbow were adjusted to match the median angles and distances identified by Mogul. These modified restraints were then applied during refinement in Coot and REFMAC.

##### Distinguishing the nucleotide status of eEF1A

As the elongation complex was isolated directly from lysate, the nucleotide status of eEF1A is undefined. However, the observed density and the nucleotide coordination environment is consistent with the cryo-EM structure of EF-Tu⋅GDP (Fischer et al., 2015) and the high-resolution crystal structure of HRas⋅GDP (Klink et al., 2006). Thus, the bound nucleotide can be confidently assigned as a GDP with a Mg ion coordinated to the β-phosphate (Figure S6A). The presence of GDP is consistent with data showing that didemnin B does not inhibit the GTPase activity of eEF1A (Crews et al., 1994), and with didemnin B sharing a mechanism, as well as a binding site, with kirromycin, which traps the GDP-bound state of EF-Tu (Schmeing et al., 2009).

The presence of GTP can be excluded, as the density is insufficient to account for a γ-phosphate and a coordinated Mg ion, which bind together in translational GTPases (Voorhees et al., 2010) (Figure S6). We can also exclude the possibility that didemnin B has trapped the state after GTP hydrolysis but prior to release of inorganic phosphate (P_i_), as related GTPases in the GDP+P_i_ state also coordinate a Mg ion (Pasqualato and Cherfils, 2005) (Figure S6A).

##### Termination complex

The model for human eRF1 (UniProt: P62495) was taken from our previous structure of the 80S⋅eRF1⋅ABCE1 complex bound to a UGA stop codon (PDB accession code 3JAI) (Brown et al., 2015b) and fitted to the 80S⋅eRF1⋅eRF3 and 80S⋅eRF1 maps. The individual domains of eRF1 were moved manually and rigid-body fitted in Coot to fit the pre-accommodated state in the 80S⋅eRF1⋅eRF3 map, while only minor modifications were necessary to model accommodated eRF1 in the 80S⋅eRF1 map. The AAQ sequence was mutated to GGQ in the 80S⋅eRF1⋅eRF3 model to reflect that this complex was reconstructed using wild-type eRF1 and not with catalytically inactive eRF1(AAQ).

A model for eRF3a (Uniprot: P15170) was constructed using the crystal structure of the human eRF1⋅eRF3 complex (PDB accession code 3E1Y) (Cheng et al., 2009) and the moderate-resolution cryo-EM structure of the mammalian 80S⋅eRF1⋅eRF3 termination complex (PDB accession code 3J5Y) (des Georges et al., 2014) as templates. GMPPCP was modeled into the active site of the eRF3 G domain.

Real space refinement was performed to optimize the fit of all eRF1 and eRF3 sidechains, as well as changes to the ribosome at the binding interfaces and decoding center.

##### Rescue complex

Models for human Pelota (UniProt: Q9BRX2) and Hbs1l (UniProt: Q9Y450) were built using the deposited models for the moderate-resolution reconstruction of Dom34⋅Hbs1 (the yeast homologs of Pelota⋅Hbs1l) bound to a ribosome stalled by a synthetic stem loop (PDB accession code 3IZQ) (Becker et al., 2011) as a template. In this reconstruction, additional density was observed at the entrance to the mRNA channel that was assigned to the N-terminal domain of Hbs1. This interaction is absent in our reconstructions, which may reflect differences in the mRNA substrates used to program stalling, or between the N-terminal domains, which in mammals and yeast share little sequence identity. Therefore, only the G domain and domains 2 and 3 of Hbs1l were modeled. GMPPCP was modeled into the active site of the Hbs1l G domain.

In the 80S⋅Pelota⋅Hbs1l complex formed with a truncated mRNA, additional density at the ‘latch’ between h18 in the body and h34 and uS3 in the neck of the SSU appears to correspond to a bound GMPPCP molecule that is probably an artifact of reconstituting the complex in the presence of 0.5 mM GMPPCP.

Real space refinement was performed to optimize the fit of all Pelota and Hbs1l sidechains, as well as changes to the ribosome at the binding interfaces and decoding center.

#### Model refinement and validation

Models were refined with REFMAC v5.8 utilizing external restraints generated by ProSMART and LIBG (Brown et al., 2015a). Model statistics were obtained using MolProbity (Chen et al., 2010). Cross-validation was calculated as previously described (Amunts et al., 2014, Brown et al., 2015a).

#### Molecular graphics

All figures were generated with Chimera (Pettersen et al., 2004) or PyMOL (Schrödinger, LLC).

### Quantification and Statistical Analysis

All reported resolutions are based on the Fourier shell correlation (FSC) 0.143 criterion (Rosenthal and Henderson, 2003).

### Data and Software Availability

#### Data Resources

Nine maps have been deposited with the EMDB with accession codes EMDB: 4129, EMDB: 4130, EMDB: 4131, EMDB: 4132, EMDB: 4133, EMDB: 4134, EMDB: 4135, EMDB:4136, and EMDB: 4137. Atomic coordinates have been deposited with the Protein Data Bank under accession codes PDB: 5LZS, PDB: 5LZT, PDB: 5LZU, PDB: 5LZV, PDB: 5LZW, PDB: 5LZX, PDB: 5LZY and PDB: 5LZZ.

## Author Contributions

Conceptualization, S.S., J.M., A.B., J.T., V.R., and R.S.H.; Investigation and Initial Draft, S.S., J.M., and A.B.; Editing, All Authors.

## Supplemental Information

Document S1. Tables S1 and S2

Table S3. 28S and 18S rRNA Sequences, Related to Figure 1
