# Supplementary material for: Decoding Mammalian Ribosome-mRNA States by Translational GTPase Complexes
Source: Cell. 2016 Nov 17;167(5):1229–1240.e15. doi: 10.1016/j.cell.2016.10.046 (PMC5119991; doi:10.1016/j.cell.2016.10.046)
Supplement: Document S1. Tables S1 and S2 [file mmc1.pdf]

Cell, Volume *167*

Supplemental Information

Decoding Mammalian Ribosome-mRNA States by Translational GTPase Complexes

Sichen Shao, Jason Murray, Alan Brown, Jack Taunton, V. Ramakrishnan, and Ramanujan S. Hegde

Table S1. Data collection and processing statistics.

	80S•P-site tRNA	80S• aa-tRNA• eEF1A	80S•eRF1• eRF3	80S•eRF1	80S•eRF1• ABCE1 (combined)	80S•Pelota• Hbs1• mRNA (truncated)	80S•Pelota• Hbs1• mRNA (stop)	80S•Pelota• Hbs1• mRNA (polyA)	80S•Pelota• Hbs1 (combined)
**Data Collection**									
Voltage (kV)	300	300	300	300	300	300	300	300	300
Pixel size (Å)	1.04	1.04	1.34	1.34	1.34	1.34	1.34	1.34	1.34
Defocus range (μm)	0.32-3.97	0.71-3.58	0.61-4.57	1.20-4.41	0.69-5.29	0.97-3.97	0.55-3.80	0.87-6.13	0.55-6.13
Defocus mean (μm)	2.15	2.21	2.33	2.68	2.75	2.48	2.20	2.50	2.36
Electron dose (e- $Å^{-2}$)	40	40	30	30	30	30	30	30	30
**Data processing**									
Independent data collections	1	1	2	1	4	1	1	2	4
Useable micrographs	1,643	1,627	2,611	1,611	5,706	1,815	1,556	1,112	4,483
Particle picking software	EMAN2	EMAN2/ RELION	EMAN2/ RELION	RELION	RELION	RELION	RELION	EMAN2/ RELION	EMAN2/ RELION
Particles picked/sorted	158,287	139,655	211,664	105,812	1,248,861	181,880	167,508	138,488	487,876
Particles after 2D classification	126,483	99,148	189,744	100,807	1,023,975	179,727	129,274	127,585	436,586
Particles after 3D classification	59,594	-	72,875	58,869	92,552	67,287	93,174	77,534	237,995
Particles after FCwSS	25,358	46,708	61,752	13,852	80,571	42,011	37,432	20,717	100,160
Final particles	25,358	40,347	61,752	13,852	80,571	42,011	37,432	20,717	58,773
Map sharpening B-factor ($Å^2$)	-51.6	-64.8	-63.3	-63.8	-66.4	-84.1	-56.8	-63.6	-63.6
Resolution	3.06	3.31	3.65	3.75	3.35	3.53	3.67	3.99	3.47
EMDB accession code	4129	4130	4131	4132	4133	4134	4135	4136	4137

Table S2. Refinement and model statistics.

	80S•aa-tRNA•eEF1A	80S•eRF1•eRF3	80S•eRF1	80S•eRF1•ABCE1 (combined)	80S•Pelota•Hbs1•mRNA(trun)	80S•Pelota•Hbs1•mRNA(stop)	80S•Pelota•Hbs1•mRNA (polyA)	80S•Pelota•Hbs1 (combined)
**Model composition**								
Non-hydrogen atoms	220,874	222,676	219,116	223,868	222,092	221,999	221,906	221,999
Protein residues	12,144	12,555	12,111	12,704	12,505	12,497	12,490	12,497
RNA bases	5,747	5,678	5,678	5,678	5,671	5,671	5,670	5,671
Nucleotide/inhibitor	GDP/ didemnin B	GMPPCP	-	-	GMPPCP	GMPPCP	GMPPCP	GMPPCP
Metals (Zn^{2+}/Mg^{2+})	8/276	8/282	8/258	8/302	8/281	8/264	8/236	8/264
**Refinement**								
Resolution (Å)	3.31	3.65	3.75	3.35	3.53	3.67	3.99	3.47
Average B factor (Å^2)	110.3	93.6	99.1	90.9	111.1	108.9	113.8	109.4
$FSC_{average}$	0.85	0.87	0.82	0.82	0.83	0.83	0.83	0.86
**R.m.s. deviations**								
Bond lengths (Å)	0.005	0.005	0.006	0.005	0.006	0.005	0.006	0.006
Bond angles (°)	1.00	0.96	1.10	0.89	1.01	0.99	1.1	1.1
**Validation**								
Molprobity score (percentile)	2.13 (100th)	1.97 (100th)	2.11 (100th)	1.86 (100th)	2.08 (100th)	2.01 (100th)	2.35 (99th)	2.01 (100th)
Clashscore, all atoms (percentile)	2.05 (100th)	1.76 (100th)	2.54 (100th)	1.47 (100th)	2.37 (100th)	1.87 (100th)	3.51 (100th)	1.87 (100th)
Favored rotamers (%)	83.5	84.0	82.8	85.9	83.1	83.5	80.6	83.5
Poor rotamers (%)	6.7	6.1	6.7	5.6	6.6	6.3	7.7	6.3
**Ramachandran plot**								
Favored (%)	93.3	93.2	93.1	94.0	93.1	92.7	90.6	93.0
Outliers (%)	0.5	0.5	0.4	0.2	0.5	0.5	1.0	0.5
**Validation (RNA)**								
Correct sugar puckers (%)	98.0	97.8	97.7	97.8	97.7	97.8	97.7	97.8
Good backbone conformations (%)	75.4	75.1	74.4	75.0	75.1	75.5	73.5	75.5
